# Decrypting distributed ledger design—taxonomy, classification and blockchain community evaluation

**DOI:** 10.1007/s10586-021-03256-w

**Published:** 2021-04-24

**Authors:** Mark C. Ballandies, Marcus M. Dapp, Evangelos Pournaras

**Affiliations:** 1 Computational Social Science, ETH Zurich, Zürich, Switzerland; 2 School of Computing, University of Leeds, Leeds, UK

**Keywords:** Blockchain, Distributed ledger, Taxonomy, Cryptoeconomic design, Token engineering, Classification

## Abstract

More than 1000 distributed ledger technology (DLT) systems raising $600 billion in investment in 2016 feature the unprecedented and disruptive potential of blockchain technology. A systematic and data-driven analysis, comparison and rigorous evaluation of the different design choices of distributed ledgers and their implications is a challenge. The rapidly evolving nature of the blockchain landscape hinders reaching a common understanding of the techno-socio-economic design space of distributed ledgers and the cryptoeconomies they support. To fill this gap, this paper makes the following contributions: (i) A conceptual architecture of DLT systems with which (ii) a taxonomy is designed and (iii) a rigorous classification of DLT systems is made using real-world data and wisdom of the crowd. (iv) A DLT design guideline is the end result of applying machine learning methodologies on the classification data. Compared to related work and as defined in earlier taxonomy theory, the proposed taxonomy is highly comprehensive, robust, explanatory and extensible. The findings of this paper can provide new insights and better understanding of the key design choices evolving the modeling complexity of DLT systems, while identifying opportunities for new research contributions and business innovation.

**Supplementary Information:**

The online version contains supplementary material available at 10.1007/s10586-021-03256-w.

## Introduction

Over 1000 systems have emerged in recent years from distributed ledger technology (DLT), raising $600 billion in investment in 2016 [75]. They power a large spectrum of novel distributed applications making use of data immutability, integrity, fair access, transparency, non-repudiation of transactions [87] and cryptocurrencies. These applications include improving supply-chains [30, 42, 44, 45], IoT [80], creating self-sovereign identities^1^ [5, 48], establishing peer-to-peer energy markets [2, 35], securing digital voting [43, 60], e-health [36, 39, 65] and enabling international financial transactions [71, 87]. The most well-known DLT system is Bitcoin, featuring a novel consensus mechanism^2^ and a cryptoeconomic design^3^ (CED), which enables untrusted parties to reach consensus [9]. Bitcoin is the first public DLT system that prevents double-spending^4^ and Sybil attacks^5^ [79].

A *distributed ledger* (DL) is a distributed data structure, whose entries are written by the participants of a DLT system after reaching consensus on the validity of the entries. A *consensus mechanism* is usually an integral part of a distributed ledger system and guarantees system reliability: all written entries are validated without a trusted third party. Distributed ledgers are designed to support secure *cryptoeconomies*, which are capable of operating cross-border, without depending on a particular political structure or legal system. These cryptoeconomies rely on digital currencies referred to as *tokens* and cryptographic techniques to regulate how value is exchanged between the participating actors [6, 16]. The options and choices of a cryptoeconomy are referred to as *cryptoeconomic design* (CED) and this plays a key role in the stability of a DLT system in terms of convergence, liveness, and fairness [9].

Nevertheless, making system design choices (e.g, on the type of consensus mechanism) in this rapidly evolving technological landscape to meet the requirements (e.g., security or performance demands) of a broad spectrum of distributed applications is complex and challenging. The lack of a common and insightful conceptual framework for DLT has been cited as a significant barrier in this regard [54, 62]. Moreover, the system configuration space of distributed ledgers and the cryptoeconomies they support is large, which has implications on the applicability as well as cost-effectiveness of DLT systems in real-world applications [87]. To date, these configurations have not been rigorously formalized to guide researchers and practitioners on how to design DLT systems [22, 23, 62, 82]. Therefore, identifying key design choices and system configurations that can differentiate distributed ledgers and guide innovation in new DLT systems can have an impact on reducing the design complexity and cost. It has been argued that this lack of a clear positioning of DLT systems leads to a fragmentation in the DLT community and a duplication of effort [76]. The significance of this challenge is reflected in the recent taxonomies of distributed ledgers [55, 76, 82, 86–88].

This paper derives a *useful*^6^ taxonomy of DLT systems from a novel conceptual architecture. This taxonomy is then utilized to classify 50 viable and actively maintained DLT systems. In contrast to earlier work, a novel evaluation methodology is employed that solicits feedback from the blockchain community and constructively uses it to validate and further improve the proposed taxonomy and classification. Moreover, the classification data are utilized to quantitatively reason about key design choices in the observed DLT systems, which then, in turn, determine a design guideline for DLT systems. To make this design guideline objective, this paper relies on systematic methods that combine in a novel way (i) literature review, (ii) novel data collection and (iii) ML-based data analysis. In particular, the data-driven approach results in a guideline that structures the modeling complexity of DLT systems and thus accelerates and simplifies the design phase by grouping together system design configurations derived from the attribute values of the taxonomy.

**Fig. 1:**
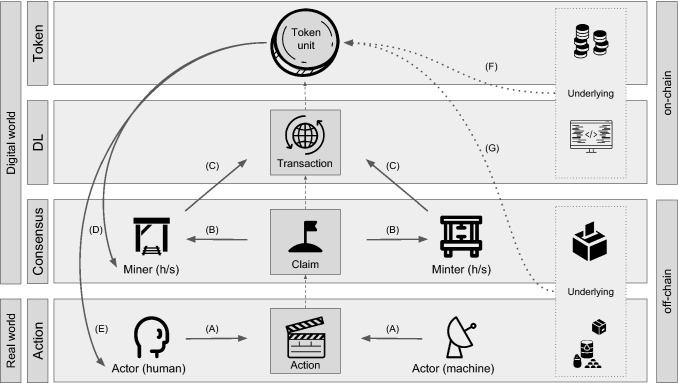
An overview of the conceptual architecture containing the four key concepts of DLT systems and their relationship: action, consensus, distributed ledger and token

**Fig. 2:**
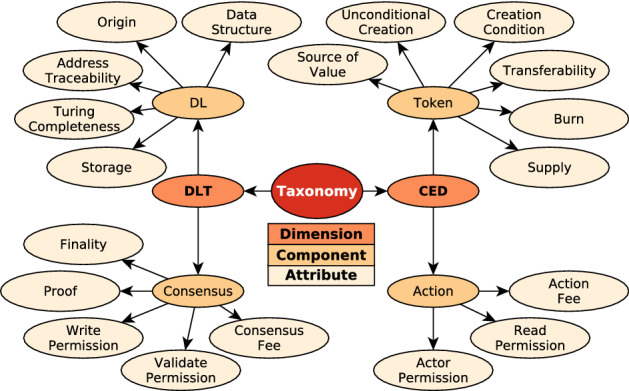
Overview of the taxonomy, depicting the two dimensions of DLT and CED, its four components and 19 attributes

**Fig. 3:**
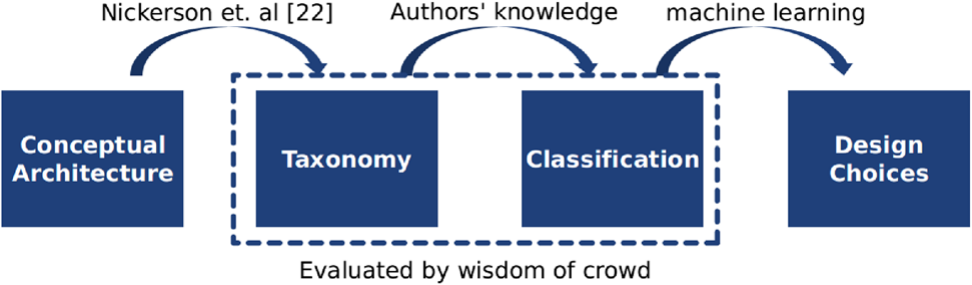
A novel methodology that combines machine learning techniques, wisdom of the crowd and taxonomy theory to reason about a DLT system design guideline

The contributions of this paper are outlined as follows:

1. *A conceptual architecture* that models DLT systems with four components. The architecture (Fig. 1) defines minimal and insightful design elements to illustrate the inner mechanics of distributed ledgers and the interrelationships of their components.
2. *A taxonomy* (Fig. 2) of distributed ledgers that formalizes a set of 19 descriptive and qualitative attributes, including a set of possible values for each attribute.
3. *A classification* of 50 DLT systems, including Bitcoin and Ethereum, backed by an extensive literature review.
4. *A taxonomy evaluation criterion* referred to as ‘expressiveness’ derived from earlier theory on taxonomies.
5. *Crowdsourced feedback* from the blockchain community to further assess and improve the taxonomy and classification.
6. *A design guideline* for DLT systems (Fig. 12), which is constructed using machine learning techniques to reason based on empirical data of viable, actively maintained and academically referenced DLT systems.
7. *A methodology* (Fig. 3) that utilizes a broad spectrum of inter-disciplinary methods to derive system design guidelines by reasoning based on machine learning techniques, wisdom of the crowd and taxonomy theory.

This paper is organized as follows: In Sect. 2, terminology and recent taxonomies for DLT systems are discussed. A conceptual architecture for DLT systems is introduced in Sect. 3, while a taxonomy is outlined in Sect. 4. Thereafter, Sect. 5 illustrates the methodology of the conducted experiments and Sect. 6 presents the evaluation. Section 7 derives based on the findings of the evaluation a design guideline for DLT systems. Finally, in Sect. 8 a conclusion is drawn and an outlook on future work is given.

## Background and Literature Review

Different types of data structures are utilized in distributed ledgers to store information. In particular, the literature distinguishes between distributed ledgers (DL) and blockchains [66, 87], the latter representing one type of data structure utilized in the former. Another type of data structure is the directed acyclic graph [46, 88].

The entries of a distributed ledger contain *transactions*. Any type of transaction can be stored, ranging from cryptographically signed financial transactions to hashes of digital assets, and Turing-complete executable programs [87], i.e. smart contracts. DLT systems often provide *access rights to these transactions*, which determine who can initiate transactions, write them to the distributed ledger, and read them again from the ledger [87]. In addition, DLT systems utilize so-called *tokens* [86], which are defined as a unit of value issued within a DLT system and which can be used as a medium of exchange or unit of account (see Sect. 4.4). These tokens span a multi-dimensional incentive system via which they can promote self-organization [41] and thus lead to benefits in society [38], such as contributing solutions for the UN Sustainable Development Goals (SDGs) [21]. Hence tokens are identified as another key component of DLT systems in addition to the distributed ledger [51]. These components can be modeled independently, resulting in systems that do not necessarily maintain a native distributed ledger. In such cases, a token is defined while another system is used to provide the infrastructure for a distributed ledger. For instance, the Aragon system does not maintain a natively developed distributed ledger [20].

The ability to define the type of transactions, access rights and tokens is used to regulate the behavior of users, i.e. by limiting and granting access rights to system services or by incentivizing specific actions with tokens. These socio-economic choices not only influence aspects of the system stability, such as the correctness, liveness and fairness of the consensus mechanism [9], but also determine how complex cryptoeconomies emerge [6, 16]. In other words, cryptoeconomic design (CED) plays a key role in enabling DLT systems to reach stability and underpin how the economies form.

A DLT system has to reach *consensus* before a transaction can be permanently written to its ledger [86]. This consensus mechanism is a functional element of any DLT system [66], as it enables a decentralized network to take decisions about the validity of entries in the distributed ledger [67]. In particular, in the context of DLT systems, consensus prevents token units from being spent twice [49] and Sybill attacks [79], which is where fake identities are used to inject false information into the distributed ledger.

### Comparison of taxonomies for DLT systems

**Table 1:** Comparative overview of earlier work outlining the landscape of distributed ledgers

1.	2.	**3.**	**4.**	**5.**	**6.**	**7.**	**8.**	**9.**	**10.**	**11.**	**12.**
ID	Paper	**Concept**	**Attributes**	**Conensus Incent.**	**Diff. DL**	**CED**	**Access rights to transactions**	**Token properties**	**Classification**	**Community evaluation**	**Quantitative analysis**
1	Tasca et al. [76]	–	30	Yes	–	Yes	Yes	Yes	–	–	–
2	Comuzzi et al. [14]	–	8	Yes	–	Yes	Yes	–	–	–	–
3	Xu et al. [87]	–	13	–	Yes	Yes	Yes	–	–	–	–
4	Xu et al. [86]	Yes	7	–	–	Yes	Yes	–	–	–	–
5	Yeow et al. [88]	–	4	–	Yes	–	–	–	Yes	–	–
6	Okada et al. [56]	–	4	Yes	–	Yes	–	–	–	–	–
7	Wieninger et al. [82]	–	11	Yes	–	Yes	Yes	Yes	–	–	–
8	Dinh et al. [23]	Yes	9	–	–	Yes	Yes	–	Yes (partial)	–	–
9	De Kruijff et al. [18]	–	6 (many)	–	–	–	–	–	–	–	–
10	Sarkintudu et al. [69]	–	5	–	–	–	–	–	–	–	–
11	Notheisen et al. [55]	–	6	–	–	Yes	Yes	–	Yes	–	–
	*This paper*	*Ye*s	*19*	*Yes*	*Yes*	*Yes*	*Yes*	*Yes*	*Yes*	*Yes*	*Yes*

Recent ontologies and taxonomies have been proposed to structure the design space of DLT systems. A comparative summary of earlier work is shown in Table 1. Column 3 of that table depicts if the paper utilizes a conceptual architecture to construct the taxonomy. Nickerson et al. [52] suggest to conceptualize the domain of interest for which a taxonomy is developed. In such a conceptual architecture, the attributes of a taxonomy should be positioned such that these are mutually exclusive and collectively exhaustive [52]. Nevertheless, only Papers 4 and 8 in Table 1 provide a conceptual architecture (Column 3 in Table 1) that determines the choice of some of the attributes. For instance, Paper 4 distinguishes between on-chain and off-chain components: attributes of the DLT system that exist on the distributed ledger (e.g. permission management) vs. attributes that exist outside (e.g. control, data).

A *useful* taxonomy should be concise and robust [52], hence using a limited number of attributes that differentiate the objects of interest. The number of attributes listed in the papers varies considerably, from 4 to 30 (Column 4 in Table 1). One explanation is that the papers focus on different aspects of DLT systems and thus study different (sub)sets of attributes. For instance, Paper 5 focuses on Internet of Things applications of DLT systems and only use four attributes (Column 4 in Table 1), whereas Paper 1 designs a taxonomy to model all types of DLT systems and hence uses 30 attributes (Column 4 in Table 1). Nevertheless, none of the papers justifies the number of selected attributes. In particular, their impact on conciseness and robustness of the taxonomy is not evaluated [52]. Also, several of the attributes potentially overlap with each other conceptually due to the aforementioned lack of a conceptual architecture.

Consensus is identified as a core feature of DLT systems [67] and as such, it is incorporated in all papers listed in Table 1. For this reason, it is omitted from this table. Nevertheless, just four papers consider schemes to incentivize participation in the consensus mechanism (Column 5 in Table 1).

Moreover, only Papers 3 and 5 distinguish between the different types of data structures in distributed ledgers (Column 6 in Table 1). For instance, Paper 3 differentiates between blockchains and directed acyclic graphs. Nevertheless, some of the most recent contributions solely include blockchain-based DLT systems [14, 76, 82, 87].

Eight papers include cryptoeconomic design in their taxonomy (Column 7 in Table 1). In particular, seven papers consider access rights to transactions (Column 8 in Table 1). Only Papers 1 and 7 derive a taxonomy that include tokens and their properties (Column 9 in Table 1).

Three papers illustrate a classification of DLT systems based on their proposed taxonomy (Column 10 in Table 1). For instance, Paper 5 illustrates the classification of 28 DLT systems. The authors rely on three attributes: data structure, scalable consensus ledger, and transaction model [88]. However, neither of the papers introduces a formal methodology to select the classified DLT systems, which lowers their objectivity. Also, without a formal selection methodology, it is not guaranteed that the taxonomy enables a comprehensive classification of all known DLT systems [52].

The *usefulness* of a taxonomy depends on qualitative criteria studied in taxonomy theory [52]. An approach to assess the usefulness of a taxonomy is to utilize crowdsourced community feedback and thus the wisdom of the crowd. This is particularly relevant in the case of DLT systems and the blockchain community. As the community shapes the blockchain landscape, soliciting their feedback can provide both, invaluable new insight into the design of DLT systems and increase the usefulness of a taxonomy. Nevertheless, such an endeavor has not been pursued until nowadays, as shown in Column 11 of Table 1.

Finally, a quantitative evaluation and analysis of taxonomy and classification elements by means of statistical or machine learning methods have not been performed so far (Column 12 in Table 1). This is a missed opportunity, as such an approach can provide more objective insights into the usefulness of taxonomies and identify key design choices in DLT systems that structure the modeling complexity of these systems at design phase, as demonstrated in this paper (Sect. 6.2).

### Summary of limitations

In summary, a few observations can be made about current DLT system taxonomies. First, they predominantly focus on the DL and consensus mechanisms, while largely missing the role of cryptoeconomics and token design, despite their significant influence on system stability [9]. Second, the interrelationships between the different components as well as the choice of attributes are usually not based on an overarching conceptual architecture. Third, only three of the papers classify DLT systems. Nevertheless, these papers neither utilize a rigorous scientific methodology nor quantitatively analyze their classification. As a result, classification is usually not formally validated and the identification of design choices is limited to qualitative criteria. Last but not least, none of the proposed taxonomies are systematically refined based on feedback from blockchain practitioners. Such a complementary external validation process promises to produce more unbiased taxonomies.

This paper addresses all of the aforementioned limitations identified in the literature and contributes a useful taxonomy as defined in earlier taxonomy theory [52], built on a solid conceptual architecture, assessed via classifications and validated by both, feedback from the blockchain community and machine learning methods. Moreover, the quantitative analysis of the classification is utilized to identify key design choices in observed DLT systems.

## Conceptual architecture

Based on the study of 50 DLT systems (see Table 2 in Supplementary Material for an overview of these systems), a conceptual architecture is introduced in this section. The architecture is composed of a set of four key components and shows, how they relate to each other as well as how they are positioned in the distributed ledger design space. The architecture is depicted in Fig. 1. The four components are illustrated in the rest of this section.

*Action component:* A human or machine performs an action in the real world (Arrow A in Fig. 1), for example planting a tree or carrying out a monetary transaction. Here, at the border between the real world and digital world, the action is represented digitally, and is referred to as claim.

*Consensus component:* Claims are broadcast to all nodes in the network that can participate in the consensus mechanism (Arrow B). These nodes (referred to as miners in Bitcoin or minters in Peercoin) collect these claims to write them to the distributed ledger.

*Distributed ledger component:* Participants in the consensus mechanism combine these claims into entries (referred to as blocks in Bitcoin) and write them to the distributed ledger (Arrow C). This representation of a claim on the distributed ledger is called a transaction. Transactions and their containing objects (e.g. smart contracts) that exist on the distributed ledger are referred to as on-chain, in contrast to off-chain objects, which exist on the Consensus or Action component.

*Token component:* The way token units are created depends on whether an incentive system is part of the DLT system. If it is, there are two options: token units are given as rewards to nodes for either participating in the consensus mechanism (Arrow D) or carrying out an action (Arrow E). While the inherent properties of such tokens (e.g. whether supply is capped or not) are determined by the design of the DLT system, the value of the token units is backed by a source of value, which are cryptoeconomic assets that reside on-chain (Arrow F, for example other tokens or executable code) or off-chain (Arrow G, for example goods, services or commodities).

*Example Ethereum:* In the case of Ethereum, one type of action involves deploying a piece of code (Arrow A in Fig. 1), such as a smart contract. These actions are collected by miners (Arrow B) and written as a block to the Ethereum distributed ledger (Arrow C). A miner who successfully writes a block obtains Ether, which refers to newly created units of a token that serves as an incentive to mine (Arrow D). The Ether token has inherent properties, e.g. it has uncapped supply. It also has value because it enables its owner to access the on-chain computational power of the Ethereum network (Arrow F).

## Taxonomy

Based on the conceptual architecture of Sect. 3, a taxonomy is designed, using the method proposed by Nickerson et al. [52]. The goal of the taxonomy is to enable a comprehensive classification of DLT systems that enable the quantitative derivation of key design choices in these systems. For this, the taxonomy illustrates both, the distributed ledger technology (DLT) and the cryptoeconomic design (CED) of academically relevant DLT systems. For this, the taxonomy positions the four components from Sect. 3 across two dimensions (Fig. 2). The first dimension concerns aspects of the system design related to distributed ledger technology (DLT)—*Distributed Ledger component*, *Consensus component*—, while the second dimension concerns aspects pertaining to cryptoeconomic design (CED)—*Action component* and *Token component*. In the following sections, the attributes of each component are illustrated in greater detail.

### Distributed ledger

#### Definition 1

A distributed ledger is defined as a distributed data structure, containing entries that serve as digital records of actions.

In the Bitcoin system, an entry in the data structure is called a block. In the IOTA system, it is called a bundle [68]. An entry contains a set of transactions (Fig. 1, DL component). In Bitcoin, these transactions represent the exchange of cryptocurrency value. The attributes of the distributed ledger are *data structure*, *origin*, *address traceability* and *Turing completeness*.

*Data structure* denotes in which format data is stored on the distributed ledger. It can be one of the following: *blockchain*, *directed acyclic graph* (DAG) or *other*. The well-known data structure is a blockchain; an immutable and append-only linked list that has a total order of elements. Several systems use blockchains, such as Bitcoin [87], Ethereum [19] and Litecoin [34]. In contrast to these systems, IOTA uses a directed acyclic graph [88]. This data structure is no longer a linked list, but a directed graph with no cycles, leading to a partial order of elements. When compared to Blockchains, DAGs trade off security (e.g. risk of double spending attack) against a higher transaction throughput by facilitating fast entry confirmation times [28]. Ripple neither uses a blockchain nor a directed acyclic graph but instead operates on other consensus-based accounting mechanism [33].

*Origin* refers to who maintains the distributed ledger. The attribute value can either be *native*, if the distributed ledger is maintained by and for the system itself or *external*, if the system uses a distributed ledger from another DLT system or *hybrid* if the systems maintain their own distributed ledger in combination with a distributed ledger of another DLT system. The level of maintenance varies between different DLT systems. Bitcoin develops and maintains its distributed ledger natively, as does NXT [86]. In contrast, Aragon [20], Augur [33, 59] and Counterparty [88] does not maintain a native distributed ledger, opting to use the Ethereum or Bitcoin infrastructure instead. Systems can use a hybrid approach. Factom combines a natively developed blockchain and its own consensus mechanism with the Bitcoin blockchain [87].

*Address traceability* denotes the extent to which different transactions that originate from or arrive at the same chain identity, can be linked together. The value can either be *obfuscatable*, if the distributed ledger has mechanisms in place to hide such links or *linkable,* if these links can be inferred with some computational effort. The level of address traceability varies between the different DLT systems. Zcash [37] and Monero [74] are so-called privacy coins, which perform advanced measures to unlink transactions [9]. Hence, the on-chain identities of the actors remain obfuscated. Bitcoin has linkable address traceability [9]. In theory, transactions cannot be linked to a particular chain identity, but it has been shown that this can actually be achieved with some computational effort [87]. The same applies to Ripple [50].

*Turing completeness* refers to whether a Turing machine can be simulated by the DL and can either be *Yes* or *No*.^7^ Some DLs, such as Ethereum, can execute Turing machines. This allows Turing complete smart contracts to be stored and executed [86], in contrast to the Bitcoin blockchain [9].

*Storage* denotes whether additional data can be stored on the distributed ledger beyond the default transaction information. The attribute value can either be *yes* if data can be stored or *no*, if additional data cannot be stored. The distributed ledger of Bitcoin allows arbitrary data to be stored inside transactions. This allows Bitcoin to be used as a base layer for other DLT systems, such as observed in the Counterparty system [88]. In contrast to Bitcoin, IOTA does not allow additional data to be stored [77].

### Consensus

#### Definition 2

Consensus is the mechanism through which entries are written to the distributed ledger, while adhering to a set of rules that all participants enforce when an entry containing transactions is validated.

The attributes of consensus are *finality*, *proof*, *write permission*, *validation permission* and *fee*. Due to the scope of the taxonomy to enable a comprehensive classification of all components of a DLT system (Fig. 1), more granular consensus attributes such as verification speed are not considered. Nevertheless, detailed consensus attributes can be found in [12, 49].

*Finality* refers to the guarantee that past transactions can not be changed or reversed. Its value is *deterministic* if consensus is guaranteed to be reached in finite time, or *probabilistic* if there is some uncertainty over whether consensus can be reached. In other terms, with regard to the CAP theorem, a deterministic consensus is consistent and a probabilistic algorithm can reach eventual consistency [17]. Byzantine Fault Tolerance (BFT) algorithms tolerate a class of system failures that belong to the Byzantine Generals Problem. In particular, a consensus algorithm with this property prevents, under some guarantees,^8^ consensus participants from writing a false entry to the distributed ledger. The classic system layout in BFT are permissioned systems, which finalize agreement on entries deterministically and are safe in asynchronous environments [13]. In contrast, Nakomoto consensus signaled a transition from these permissioned systems to permissionless systems that only give probabilistic guarantees about entries in a distributed ledger [57]. This type of algorithm validates each new entry using the entire history of previous entries: An entry is accepted if there is a certain number of new entries referencing it [9]. For instance, in the case of Bitcoin, a writer validates a transaction by considering the whole blockchain and then including the transaction in a new block. When this block is referenced by six other blocks, it is confirmed, as the probability that a second chain of six blocks referencing each other, but not referencing this block, is low [87], thus leading to eventual consistency [17]. Similarly, the directed acyclic graph of IOTA confirms an entry when it is referenced by a significant number of new entries [88]. On the other hand, Ripple does not use a Nakamoto consensus algorithm and it is guaranteed that consensus can be reached in a finite period of time [67].

*Proof* is the evidence used to achieve consensus. The value can either be *proof-of-work* (PoW), if consensus is achieved using the processing power of computers; *proof-of-stake* (PoS), if it is achieved through voting processes linked to (economic) power in the system; *hybrid*, if it is a combination [11] of the previous two or *other*, if another form of proof is required. Participants in the consensus mechanism require proof before accepting the validity of an entry. Bitcoin uses a proof-of-work [86], which is the solution to a mathematical puzzle that requires computational processing power and which thus mitigates the risk of Sybil attacks by linking the power of creating new entries with computational work to be performed [73]. A proof-of-stake is used by Ardor [76], which is the approval of a randomly selected consensus participant who must hold a stake in Ardor token units. This proof mitigates the risk of Sybil attacks by linking entry creation power to the economic value of hold tokens [10].

*Write permission* denotes who is allowed to write entries to the distributed ledger. The value can either be *restricted*, if participation is restricted or *public*, if it is not. Besides the CAP Theorem, a tradeoff between decentralization, consistency and scalability (DCS) can be observed in DLT systems [89]. Likewise to the CAP theorem, only two of the properties can hold simultaneously. In context of write permission, a restricted access impacts decentralization negatively and performance positively [89]. In particular, a restricted write permission can facilitate the deployment of efficient consensus protocols such as Practical Byzantine Fault Tolerance (PBFT) [73] which can mitigate Sybil attacks more efficiently when compared to proof-of-work algorithms by whitelisting and bounding consensus participants to behave correctly via contractual obliagations [73]. The Bitcoin consensus mechanism is public [86], meaning that it allows everyone who has computing power to participate [67] resulting in a system characterized by high decentralisation [89] that mitigates Sybil attacks via its proof-of-work (Section 4.2). Conversely, the consensus mechanism of Ripple is restricted [86], meaning that only a few trusted institutions can participate [88] resulting in a centralized system with a higher performance in terms of transaction throughput.

*Validate permission* signifies who is allowed to validate claims before they are written to the distributed ledger. The value can either be *restricted*, if participation is restricted or *public,* if it is not. As for the write permission, a restricted validate permission impacts decentralization negatively and performance positively. In the case of Bitcoin, writers validate the correctness of claims before writing them to a block: hence, the validation permission is public. In contrast, in the case of IOTA, a central entity, the coordinator, validates transactions before they are collected in an entry and written to the directed acyclic graph [88] resulting in a higher scalability of the system.

*Fee* denotes whether participants in the consensus (writers and validators) are paid a fee for validating new entries and writing them to the distributed ledger. The value can either be *yes* or *no*. In contrast to Bitcoin, where writers/validators are rewarded with fees [67], IOTA writers and validators receive no fees [88]. In the case of Ripple, consensus participants are not rewarded with fees, although actors need to pay a fee [64]. This system layout is captured by the fee attribute in the Action component (Sect. 4.3).

### Action

#### Definition 3

An action is one or more real-life activities that can be digitally represented in a DLT system as a transaction.

In this sense, a transaction represents a real-life action digitally. Attributes that illustrate the access rights to and the cost associated with these digital representations are *actor permission*, *read permission* and *fee*.

*Actor permission* denotes who can perform an action. The value can either be *restricted* if actors have to fulfill special requirements before performing actions or *public*, if anyone can perform actions. Bitcoin allows everyone to create a private key to send and receive token units [76]: hence, it has a public actor permission. Ripple uses restricted access rights. In order to comply with regulations (e.g. know-your-customer), actors need to register [76].

*Read permission* refers to actors that can read the contents of transactions from the distributed ledger. The value can either be *restricted*, if preconditions need to be fulfilled before permission is granted, or *public*, if permission is not restricted. Most DLT systems have public read access in the sense that everyone can read the content of the actions, which have occurred, e.g. the number of bitcoins transferred [76]. Systems utilizing privacy coins often restrict read access to the actors involved in a transaction (e.g. Zcash [87]), usually by making an effort to hide the number of token units transferred [9].

*Fee* denotes whether an actor has to pay a fee for performing an action that is unrelated to the consensus. The values are *yes* or *no*. Some DLT systems require actors to pay a fee that is unrelated to the consensus before they can store an action on the distributed ledger. For instance, actors have to pay a fee in Augur, which is not distributed to consensus participants [59] but given to actors providing services in the system. In the case of Bitcoin, no additional fee is required to perform an action, except the fee paid to the consensus participants. Ripple also requires actors to pay a fee for each action, which is not paid to consensus participants but is subsequently destroyed [64].

### Token

#### Definition 4

Token is a unit of value issued within a DLT system and which can be used as a medium of exchange or unit of account.

The associated attributes are *supply*, *burn*, *creation condition*, *unconditional creation* and *source of value*.

*Supply* refers to the total quantity of token units made available. The value can either be *capped*, if the total supply is limited to a finite number or *uncapped* otherwise. If demand increases for a token, a capped supply can cause the perceived token value to appreciate and corresponds to a deflation in prices nominated in this token. Moreover, it can result in an appreciated exchange rate with other tokens, which in turn, increases the stability of a DLT system [9]. Bitcoin has a capped supply of 21 million units [76], whereas Dogecoin does not have an upper limit [9].

*Burn* denotes whether token supply is reduced by removing token units. The values are *yes* or *no*. Some DLT systems destroy token units in a process referred to as ‘burn’. If demand remains constant, this decrease in the money supply causes token units to appreciate and hence, results in a better exchange rate with other tokens. For example in the case of Ripple, paid fees are removed from the total supply and are not returned [64]. In contrast, Bitcoin has no inherent mechanism to destroy token units.

*Transferability* refers to whether the ownership of a token unit can be changed. The value can either be *transferable*, if the token can be transferred, or *non-transferable* otherwise. Bitcoin token units can be transferred between different actors. Akasha plans to use non-transferable reputation tokens, so-called Mana and Essence [8].

*Creation condition* denotes whether the creation of new token units is linked to the incentivization of the consensus mechanism and/or an action. The value can either be *consensus*, if creation is linked to the consensus mechanism, *action*, if creation is linked to an action, *both*, if creation is linked to the consensus mechanism as well as an action, or *none* otherwise. In the case of Bitcoin, new tokens are created to incentivize the consensus mechanism [87]. Other systems create new tokens to incentivize an action. For instance, Steemit creates new steem to incentivize content creation on the platform (e.g. writing blog articles) [72]. Moreover, Ripple does not use its token to incentivize the consensus mechanism or an action [64]. Furthermore, hybrid versions are possible, where new tokens are created to incentivize both the consensus mechanism and an action. For instance, newly created token units in the DASH system are awarded to both the consensus participants and the master nodes, who perform actions such as mixing transactions to enable obfuscatable address traceability [15].

*Unconditional creation* refers to the number of new token units that can be created that do not serve to incentivize the consensus mechanism or an action. The value can either be *partial*, if some tokens are created unconditionally, *all*, if all tokens are created unconditionally (e.g. 100 % pre-mined tokens), or *none* otherwise. At the genesis of the Bitcoin system, no token units had previously been mined and all tokens come into existence by incentivizing the consensus [9]. On the other hand, all Ripple tokens were created during the genesis of the system. In the case of Augur, some tokens were created during the genesis of the system [59].

*Source of value* denotes the source of a token value and what it consists of. The value can either be *token*, if the token grants access to another token; *distributed ledger* if the token grants access to the distributed ledger, e.g. if the token is needed in order to use the storage or computing capacity of the distributed ledger; *consensus*, if the token grants access to the consensus mechanism, e.g. in a proof-of-stake type system; *action*, if the token grants access to perform or receive actions, goods or services in the real world; or *none*, if the token has no source of value. The first two values (distributed ledger and token) are considered to be on-chain and the latter two are considered to be off-chain source of values of a token unit (as depicted in Fig. 1). The Ethereum token allows everyone to store data or smart contracts on-chain [87] and to access in this way the distributed ledger of the network. Hence, the source of value of Ether token units is that they grant access to the processing power of the distributed ledger. In contrast to Ether, the Golem network token units allow holders to access off-chain computations [33]. Thus, its source of value is action as the token provides access to a service in the real world (Action component). Siacoin enables the storage of arbitrary data on both its distributed ledger [70] and its off-chain network [83]. Hence its source of values reside in the DL and Action components.

### Classification of recent DLT based distributed computing Systems

Table 2 depicts six recent works in the 19 attributes: These contributions focus on blockchain-based systems and are not utilizing other structures such as directed acyclic graphs (DAG) . Moreover, none of the systems utilizes a cryptoeconomic token. Li et al. [47] state that creating the economic model for such a mechanism is a complex design problem. Two systems develop a native distributed ledger, four systems use the computing power of the Ethereum blockchain and one system takes a hybrid approach by combining a native blockchain implementation with Ethereum in their system. No other DLT system such as Bitcoin or NEO is utilized as a first layer system, despite them being used in viable and actively maintained DLT systems (Table 4 in Supplementary Material). Finally, the documentation of the created DLT systems is in some of the recent contributions not sufficient to identify all attributes which limits the understanding of the systems design and positioning in the existing DLT system landscape. In particular, this lack of positioning could lead to a fragmentation in the DLT community and a duplication of effort [76].

**Table 2:** Classification of recent blockchain-based distributed computing systems in the 19 attributes of the introduced taxonomy

Attribute	Khalid [39]	Li [47]	Rosa [65]	Gonzalez [24]	Singh [71]	Latif [45]
*Origin*	External: Ethereum (Emulated)	Hybrid: Ethereum (Emulated)	Native	External: Ethereum	Native	External: Ethereum
*Data structure*	–	Blockchain	Blockchain	–	Blockchain	–
*Address Trace.*	–	Not specified	Traceable	–	Traceable	–
*Turing Compl.*	–	Not specified	No	–	No	–
*Storage*	–	Yes		–	Not specified	–
*Finality*	–	Probabilisitc	Probabilistic	–	Deterministic	–
*Proof*	–	Not specified	Not specified	–	PoS	–
*Write Perm.*	–	Restricted	Restricted	–	Restricted	–
*Validate Perm.*	–	Restricted	Restricted	–	Restricted	–
*Consensus Fee*	–	Not specified	Not specified	–	No	–
*Actor Permission*	Public	Not specified	Restricted	Public	Restricted	Restricted
*Read Permission*	Public	Not specified	Restricted	Restricted	Not specified	Public
*Action Fee*	No	Yes	Not specified	No	No	No
*Supply*	–	–	–	–	–	–
*Burn*	–	–	–	–	–	–
*Transferability*	–	–	–	–	–	–
*Creation Cond.*	–	–	–	–	–	–
*Uncond. Creation*	–	–	–	–	–	–
*Source of Value*	–	–	–	–	–	–

## Experimental methodology

This paper relies on a novel methodology (Fig. 3) that combines a broad spectrum of inter-disciplinary methods to contribute a useful and practical design guideline for the DLT community: Based on the introduced conceptual architecture (Fig. 1) a taxonomy is derived by the method of Nickerson et al. [52]. Utilizing the taxonomy (Fig. 2), 50 DLT systems are classified. The taxonomy and classification are evaluated by (i) the blockchain community via a survey and (ii) a quantitative analysis of real-world data. Furthermore, the quantitative analysis of the classification by the means of machine learning methods identifies key design choices in the observed DLT systems that structure modeling complexity at design phase. The design choices facilitate the construction of the design guideline. In the following, the methodologies of the classification (Sect. 5.1), the blockchain community feedback (Sect. 5.2) and the machine learning analysis (Sect. 5.3) are illustrated.

### Classification

The scope of the classification is to comprehensively capture the CED and DLT (Fig. 2) of viable, academically referenced and actively maintained DLT systems. Moreover, the classification aims at capturing the current state of DLT systems. In particular, features that are about to be released in the future are not considered. Finally, in the case that a system is 1st layer (utilizing a native distributed ledger, e.g. a mainchain) and 2nd layer (utilizing an external distributed ledger, e.g. sidechains), only the 1st layer is classified. Likewise, if a system utilizes more than one token, only the main token is classified.

**Fig. 4:**
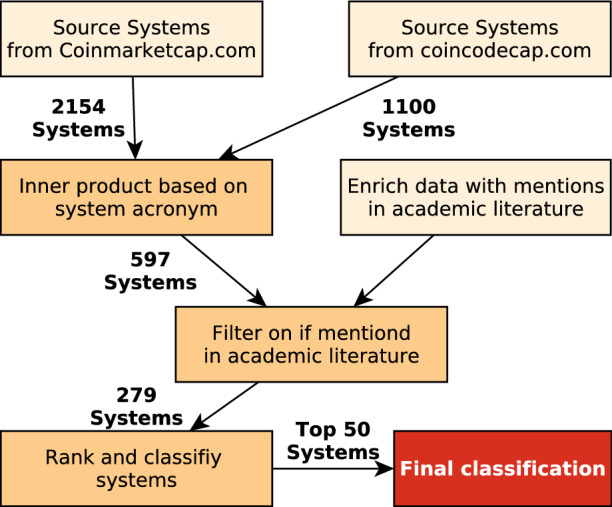
Identification and selection process of top 50 systems for classification ranked according to Sect. 5.1. The final classification is provided in the supplementary materials

In order to guarantee reproducibility, objectivity, and comprehensiveness, a system selection process for the classification is designed. Figure 4 depicts this process and visualizes the number of remaining systems per refinement step. Two websites are used:

- *Coinmarketcap.com*: Lists DLT systems ranked by their market capitalization. The rationale is that the economic value of a system is a good proxy for its viability.
- *coincodecap.com*: This site lists Github indicators of DLT systems. In particular, it contains information about the number of code commitments, Github stars, and contributors to a DLT system. These indicators capture an active development of a system.

The limitation of these data sources is that they only list systems that maintain a native cryptoeconomic token. Hence, Blockchain-as-a-Service systems,^9^ such as Hyperledger Fabric [4] are not considered. Moreover, depending on the development strategy of a system, commits might be merged externally and only pushed occasionally as major updates to Github. This may result in a lower rank of a DLT system, despite being actively maintained. This limitation is considered in the proposed ranking function (Equation 1).

Snapshots of the sources were taken on the 17th April 2019 and are merged based on the systems acronym.^10^ In order to account for academic relevance, the selection of the systems is enhanced with the number of mentions of DLT systems in Elseviers ScienceDirect database^11^ and then filtered based on the criterion of whether systems are actually mentioned in literature ($\#mentions > 0$). For the database search, the following search string is utilized on the API field *qs*:^12^ “PROJECT NAME” AND (Blockchain OR Ledger).

The remaining systems are ranked based on the following ranking function

1 $$r(i)= 0.6 \times \mathsf {m}_{\mathsf {cap}}\mathsf {(i)} + 0.3 \times \mathsf {c}_{\mathsf {commit}}\mathsf {(i)} + 0.1 \times \mathsf {c}_{\mathsf {contr}}\mathsf {(i)}$$

where $\mathsf{m}_{\mathsf{cap}}$ is the rank based on the market capitalization of a system *i*, $\mathsf {c}_{\mathsf {commit}}$ the commitment rank and $\mathsf {c}_{\mathsf {contr}}$ the contributers rank. The weights are chosen to account for the limitation of the Github activity to be a proxy for active system maintenance, hence the lower weights. The top 50 systems are then classified, based on an extensive literature review performed by the first author and checked independently by the co-authors and the blockchain community. Sources for the classification are academic literature, DLT systems websites, and whitepapers. An overview of the final classified systems can be found in Table 2 of the Supplementary Material. Moreover, the actual classification of the systems is provided in Tables 4-7 of the Supplementary Material.

### Blockchain community feedback

Participants were invited based on their contributions to Github^13^ repositories of DLT systems and their official websites. Participants received a personalized email invitation (Figure 1 in Supplementary Material) to participate in a scientific survey to rate the classification of their DLT system and the expressiveness (as defined in Sect. 6.1.3) of the proposed taxonomy. A total of 326 invitations were sent and 85 practitioners in the field responded (response rate $26.1\%$). 50 respondents completed the survey (completion rate $58.8\%$). Only completed surveys are considered in the analysis. The participants were recruited during two phases each lasting two months: The first beginning on the 22nd of March 2018 and the second on 24th July 2019. The feedback of the first phase resulted in improvements of the taxonomy, as illustrated in Sect. 2 of the Supplementary Material, and the feedback of both phases resulted in improvements of the classification.

#### Classification

In the first part of the survey, the participants were shown the classification of the four components and 19 attributes of the DLT system to which they contribute. Consult Fig. 2 for an overview of the attributes and Tables 4-7 of the Supplementary Material for the classification ratings. The participants had the option to agree, disagree, or state that they were uncertain about the classification. They could always comment on their decision, irrespective of their choice.

In order to calculate the consistency with which participants rated the classification of the same system, the consistency per attribute is calculated as follows: Assuming equidistance in the Likert scale [53], the participant responses are represented by a linear scale whereby 0 denotes disagreement, 0.5 denotes uncertainty, and 1 denotes agreement. Then, for each DLT system from which more than one response was obtained, as illustrated in Table 3, the consistency of responses is calculated for each system and attribute with the mean absolute error between the responses. Then, the average consistency for each attribute over all DLT systems is obtained by calculating the weighted average value of the previously calculated mean absolute errors.

#### Taxonomy

In the second part of the survey, the blockchain community is asked to evaluate the taxonomy (Fig. 2). Nickerson et al. propose five criteria to assess the *usefulness* of a taxonomy [52]. Namely, a taxonomy is

- *concise*, if it uses a limited number of attributes,
- *robust*, if it uses enough attributes to clearly *differentiate* the objects of interest
- *comprehensive*, if it can *classify* all known objects within the domain under considerations,
- *extensible*, if it allows for inclusion of additional attributes and attribute values when new types of objects appear,
- *explanatory*, if it contains attributes that do not model every possible detail of the objects but, rather, provide useful explanations of the nature of the objects or help to understand future objects.

The literature review (Sect. 2) reveals differences regarding how many attributes should be included in a *robust* taxonomy of DLT systems. Also, the scope of the classification is to *comprehensively* classify the CED of all academically relevant systems. Thus, considering these two points, the taxonomy is evaluated using the robustness and comprehensiveness criteria of Nickerson et al. [52]. To this end, this paper introduces the concept of expressiveness:

##### Definition 5

A taxonomy is expressive when it is robust and comprehensive.

where a robust and comprehensive taxonomy are given by Nickerson et al. [52]. The perceived expressiveness of the developed taxonomy can be determined by asking the survey participants:

##### Question 1

How *expressive* is [component/attribute] to *differentiate* between and *classify* DLT systems.

This formulation neither exposes survey participants to the theory of expressiveness, comprehensiveness, and robustness nor overloads them with a high number of questions.

The consistency calculation for the taxonomy feedback follows along the lines of the classification (Sect. 5.2.1): Despite utilizing a five-point Likert scale (from very non-expressive to very expressive) to create values ranging from zero to one, the calculation of consistency remains the same as the one for the classification.

### Machine learning analysis

In order to extract the key design choices from the classified DLT systems, two state of the art unsupervised machine learning methods are applied to the classified systems. Because the data is not labeled, supervised methods such as logistic regression are not utilized that would require access to such appropriate training data:

1. *Mulitple Correspondence Analysis* (MCA) is a statistical method that is widely used in the social sciences and which is applied in recent machine learning contributions [61, 78]. It can analyze data without a priori assumptions concerning the data, such as data falling into discrete clusters or variables being independent [1, 25]. It is a generalization of the principal component analysis (PCA) for categorical data coded in the form of an indicator matrix or a Burt matrix [26], which aims at summarizing underlying structures in the fewest possible dimensions [85]. In particular, MCA identifies new latent, pair-wise orthogonal dimensions, which are a combination of the original dimensions. [27]. Similar to PCA, these dimensions are ordered by their power to explain the amount of variance in the data [1].
2. *Kmeans* [31] for varying *k* is applied on the classification to cluster the DLT systems based on their attribute values. Clustering is a universal machine learning technique, broadly utilized in data mining [32]. The optimum number of clusters is derived by both, performing a bootstrap evaluation that determines the stability of the clusters [29] and by two well-known cluster evaluation metrics: Silhouette and Calinski-Harabasz [63].

MCA is utilized in the machine learning analysis of Sect. 6.2 to identify underlying design choices in the classified systems because it can reduce the complexity of the taxonomy to fewer dimensions by clustering the original attributes. This is an advantage of this method when compared to other standard unsupervised methods such as hierarchical clustering. K-means is then applied to validate the identified design choices. The significance of this approach lies in the fact that the design choices are derived quantitatively by reasoning based on validated empirical data: the viable and actively maintained DTL systems classified according to the taxonomy (Sect. 5.2.1).

## Experimental evaluation

The evaluation aims to identify key design choices that govern the modeling complexity of DLT systems at design phase. In order to base these insights on a strong footing, first, the taxonomy and classification are validated by feedback from the blockchain community (Sect. 6.1). Then two machine learning methods are applied on the classification to mine the design choices on a quantitative basis. (Sect. 6.2).

### Blockchain community feedback

The taxonomy (Sect. 4, Fig. 2) and classification (Table 4-7 of the Supplementary Material) are evaluated using feedback from the blockchain community.

#### Demographics

Table 3 shows the demographics of the survey participants. In particular, it shows participants specific roles for the systems and their experience. The 50 participants work in (core) technical (25 developers) and strategic (7 Project leads) positions. Moreover, 15 participants have more than 3 years of experience, 29 participants have worked 1 to 3 years, and 6 participants have worked for less than a year in the field of DLT systems. Moreover, Table 3 illustrates that the participants are involved in 33 out of the 50 classified systems.

**Table 3:**
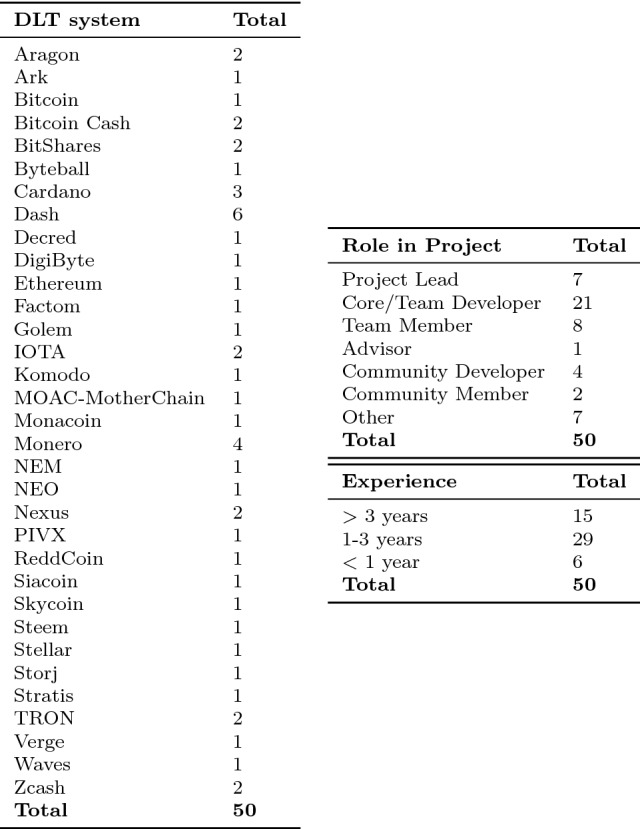
Survey participants per DLT system, their specific roles, and experience

#### Classification

**Fig. 5:**
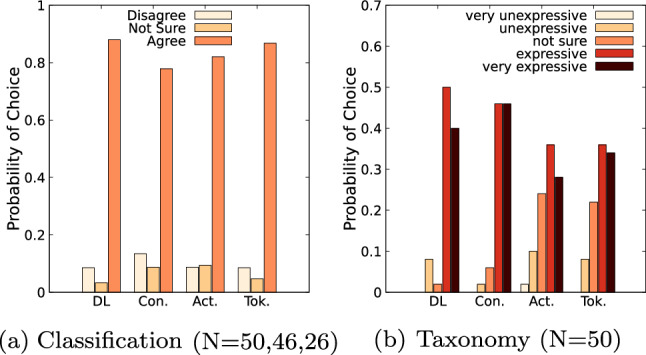
Acceptance level of the classification and expressiveness of taxonomy components as perceived by survey participants

Figure 5a depicts the aggregate acceptance level for each of the components. The Distributed Ledger component received the highest acceptance level with $88.0\%$, followed by the Token component ($86.8\%$), Action component ($82.0\%$) and Consensus component ($77.8\%$).

**Fig. 6:**
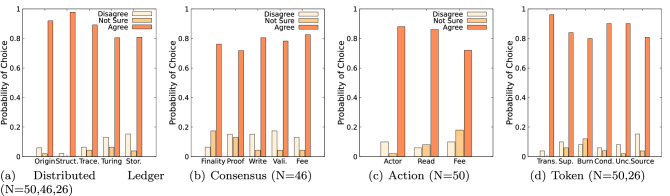
Classification evaluation of the attributes, grouped component-wise

**Fig. 7:**
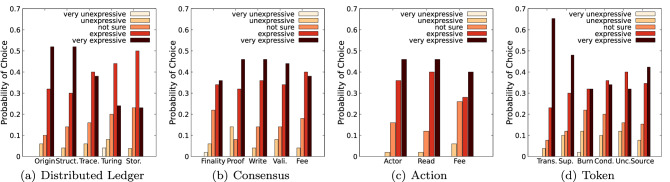
Expressiveness evaluation of the attributes, grouped component-wise (N = 50)

Figure 6 illustrates the acceptance level for each attribute of the four components. It is noteworthy that the average approval rating over all components is $83.7\%$. Five attributes are above $90\%$: transferability ($96.2\%$), origin ($92.0\%$), DL data structure ($97.8\%$), creation condition ($90.0\%$) and unconditional creation ($90.0\%$). The figure shows that the highest disagreements relate to the validate permission ($17.4\%$), source of value ($15.4\%)$ and storage ($15.4\%)$. The highest degree of uncertainty is expressed regarding the action fee ($18.0\%$), consensus finality ($17.4\%$) and consensus proof ($13.0\%$) attributes.

**Fig. 8:**
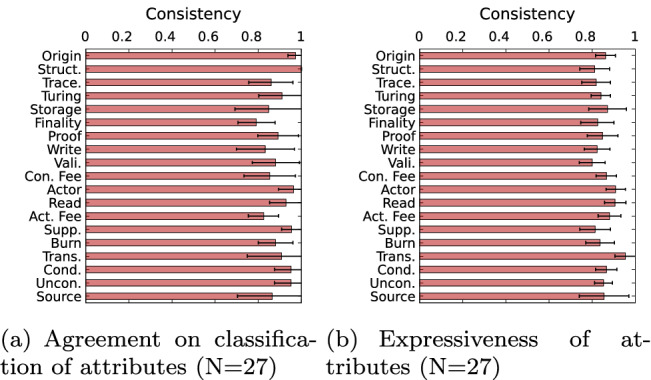
Weighted average of consistency calculation per attribute, using DLT systems consistency values of which more than one response is obtained

In order to investigate the consistency of the responses, the weighted consistency averages for each attribute are depicted in Fig. 8. The overall consistency is on average $89.9\%$. The lowest consistency measured relates to the consensus type ($79.2\%$) and action fee ($82.4\%$), correlating with the higher degree of disagreement observed earlier. The highest consistencies are observed for the DL data structure ($100.0\%$), origin ($97.3\%$), actor permission ($96.4\%$), supply ($95.8\%$), creation condition ($95.6\%$) and unconditional creation ($95.6\%$) attributes.

In a nutshell, the acceptance level of $83.7\%$ over all components and the average consistency of $89.9\%$ indicates the acceptance of the classification by the community.

#### Taxonomy

Figure 5b depicts the expressiveness of the four components as perceived by the survey participants. The Consensus component is seen as the most expressive ($92.0\%$), followed by Distributed Ledger ($90.0\%$), Token ($70.0\%$) and Action component ($64.0\%$). The highest uncertainty relates to the Action ($24.0\%$) and Token ($22.0\%$) components. The Action component consists of the lowest number of attributes, which may decrease its perceived expressiveness. In particular, the reduced number of attributes seems to hinder differentiation between DLT systems. Moreover, the literature review reveals, that Consensus is included in all taxonomies (Sect. 2). Thus this component might have been the most familiar to the participants resulting in higher expressiveness.

15 participants commented on the expressiveness of the components. They stated that a component depicting the governance of a system should be illustrated by the taxonomy ($26.6\%$),^14^ including the funding of a DLT system. Three participants ($20\%$) mention that the Action component is not expressive enough to illustrate specific features of a system, such as the distribution of actors. Similar statements were made about the Token component ($20.0\%$). In particular, it has been stated, that inter-token dynamics should be covered and that further attributes are required to illustrate the creation conditions and 1^st^ and 2nd layer tokens ($20.0\%$). Moreover, the quality of code implementation, type of programming language, strategy of code development and scalability of the system has been mentioned ($26.6\%$) as expressive attributes missing in the taxonomy. One participant stated, that the source of value attribute should be more sharply defined,^15^ and another used the opportunity to further elaborate on the system functioning. Finally, some participants made statements endorsing the construction of the taxonomy ($13.3\%$).

Figure 7 depicts the perceived expressiveness of the 19 attributes. The five most expressive attributes are deemed to be transferability ($88.5\%$), read permission ($86.0\%$), origin ($84.0\%$), actor permission ($82.0\%$), write permission ($82.0\%$) and DL data structure ($82.0\%$). Action fee ($26.0\%$), storage ($23.1\%$), consensus type ($22.0\%$) and burn ($22\%$) raise the highest degree of uncertainty. The least expressive attributes are deemed to be the consensus proof ($14.0\%$), burn ($14.0\%$) and Turing completeness/unconditional creation (each $12.0\%$) attributes. Despite the Action component being the least expressive component, two of its attributes are amongst the top five most expressive attributes. This supports the consideration to extend the action component by adding further attributes. A similar observation is made for the Token component: transferability is the most expressive attribute, but the perceived expressiveness of its component is lower than for the DL and Consensus components, which suggests extending the attributes of the Token component.

The assessment of the feedback regarding the attributes provided by the survey participants during the first recruitment phase lead to an inclusion of further attributes into the taxonomy. The nature and reasoning of these adjustments can be found in Sect. 2 of the Supplementary Material. This inclusion of new attributes indicates that the taxonomy is extensible [52].

Figure 8b depicts the consistency with which the participants evaluated the expressiveness of the taxonomy attributes. The average consistency over all attributes is $85.5\%,$ meaning that survey respondents from the same DLT systems rated the expressiveness of the taxonomy similarly to each other. In particular, they diverge from each other just $14.5\%$ on average, that is less than one choice difference on the aforementioned Likert scale.

In a nutshell, the average expressiveness rating of $79\%$ over all components and the average consistency of $85.5\%$ indicates that the taxonomy is expressive.

### Machine learning analysis

The multiple correspondence analysis is utilized to identify underlying design choices in the classified systems. In particular, the method identifies new latent dimensions, which are a combination of the original attributes of the taxonomy. In Table 4 these twelve latent dimensions and their contribution to the explained variance in the data after applying Benzceri (optimistic) and Greenacre (pessimistic) corrections are depicted in decreasing order of importance. The first four dimensions account for $96.2\%$ of total variation (for the Benzecri correction) and thus are considered significant to explain the variance in the data.

**Table 4:** Eigenvalues and corresponding explained variances of MCA dimensions after applying Benzecri and Greenacre correction

Dim	Description	Eigenvalue	Corrected variances	
			*Benzceri*	*Greenacre*
1	Layering	0.311	0.764	0.679
2	Participation	0.060	0.148	0.132
3	Staking capability	0.013	0.032	0.029
4	Cryptoec. complexity	0.007	0.018	0.016
5		0.006	0.014	0.012
6		0.003	0.008	0.007
7		0.002	0.006	0.005
8		0.002	0.004	0.004
9		0.001	0.003	0.002
10		0.001	0.002	0.001
11		0.001	0.001	0.001
12		0	0.001	0.000
13		0	0	0

**Fig. 9:**
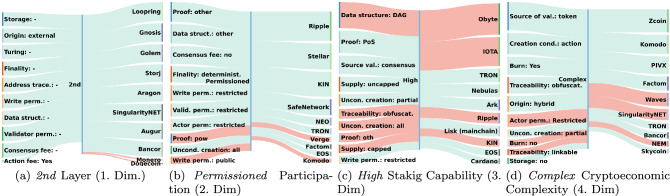
Absolute contribution [1] (flow’s thickness) of attribute values (left) and systems (right) to value of new dimension (middle/italics underneath the figures). The color code depicts if attribute value/system contributes negatively (red/dark) or positively (green/light)

Figure 9 depicts how these four dimensions are determined by both, the original attribute values of the taxonomy and the classified 50 systems. The contributions are calculated by dividing the factor scores of attributes/classified systems for a dimension by the eigenvalue of that dimension [1]. The four significant dimensions in the new vector space are in descending order of explained variance:

- *Dimension 1*: Illustrates if a system is layered. In particular, if the system uses a native distributed ledger or an external one and thus corresponds to the origin attribute of the taxonomy.
- *Dimension 2*: Illustrates the participation level in a system. In particular, the degree of openness is represented ranging from *permissioned* (e.g. restricted Actor permission) to *permissionless* systems.
- *Dimension 3*: Illustrates the capability to stake, e.g., if the system utilizes a PoS typical layout such as a token providing access to participate in the consensus.
- *Dimension 4*: Illustrates the level of cryptoeconomic complexity. The values range from complex (e.g. token interactions) to simple (e.g. tokens not burnable).

The second, third and fourth dimensions are not trivially determined by studying the classified systems visually, as the determining attribute values span over several components. Moreover, the differentiation between permissioned and permissionless systems [81, 84] and the degree of staking capability [7, 40] reflect ongoing discussion of the community on the effective design of DLT systems. The actor permission attribute contributes significantly to the construction of the permissionless dimensions, as depicted in Fig. 9b, and hence this dimension extends the permissionless concept from the consensus to the Action component. Neither Bitcoin nor Ethereum contributes significantly to the construction of the new dimensions, despite being studied the most in academic literature (588, respectively 296 citations in Elsevier’s ScienceDirect database). This might be due to other systems adopting the design of these well-known DLT systems and hence their design does not contribute significantly to the variance in the data. Additionally, observing the systems that contribute the most to the 4th dimension (level of cryptoeconomic complexity), one notices that these are systems that address a specific domain, respectively address a particular challenge and hence require an elaborated CED (e.g., PVIX and Zcoin are privacy chains, and Komodo and Bancor are decentralized exchanges).

**Fig. 10:**
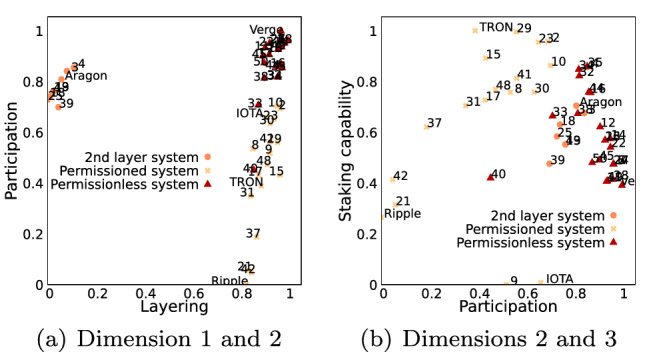
DLT systems in the latent dimensions, as identified by MCA. The labels are determined by the k-means clustering algorithm. The translation of the identifiers to DLT systems can be found in Table 1 of the Supplementary Material. Moreover, Fig. 3 in the Supplementary Material illustrates other combinations of dimensions

Figure 10 depicts the 50 DLT systems in the new dimensions. A strong clustering of systems can be observed for the first two dimensions (Fig. 10a, and a weaker for the 2nd and 3rd dimensions (Fig. 10b, which is explained due to to the lower explained variance in the data by the latter dimension.

Table 5 outlines the cluster stability and the number of dissolved clusters when applying k-means for various *k* on the classified attribute values of the 50 DLT systems. Comparing the bootmean^16^ (cluster-wise average Jaccard similarity) and bootbrd (cluster-wise number of times a cluster is dissolved) identifies three clusters as the most stable separation of the classification. This is further validated by the Silhouette and Calinski-Harabasz score, which identify two or three clusters to be optimal, as depicted in Fig. 4 of the Supplementary Material.

**Table 5:** Bootstrap statistics of identified clusters when applying kmeans with varying *k* on classification: $k=3$ results in the most stable clusters

k	Boot	Cluster					
		1	2	3	4	5	6
2	Mean	0.91	0.95	–	–	–	–
	brd	12	1	–	–	–	–
3	Mean	0.96	0.97	1	–	–	–
	brd	0	0	0	–	–	–
4	Mean	0.75	0.91	0.99	75	–	–
	brd	19	1	1	21	–	–
5	Mean	0.71	0.64	0.43	0.62	1	–
	brd	25	32	80	25	0	–
6	Mean	0.82	1	0.70	0.65	0.5	0.64
	brd	19	0	23	33	68	44

In Fig. 10, the DLT systems are labeled based on these clusters. One notices, considering the distribution of labels in Fig. 10a, that the three clusters can be identified as 2nd layer systems, permissioned systems, and permissionless systems. Likewise, utilizing the same labeling in Fig. 10b, it is noticed that these three clusters form distinct groups: 2nd layer systems being in the center, followed by permissionless and permissioned systems.

**Fig. 11:**
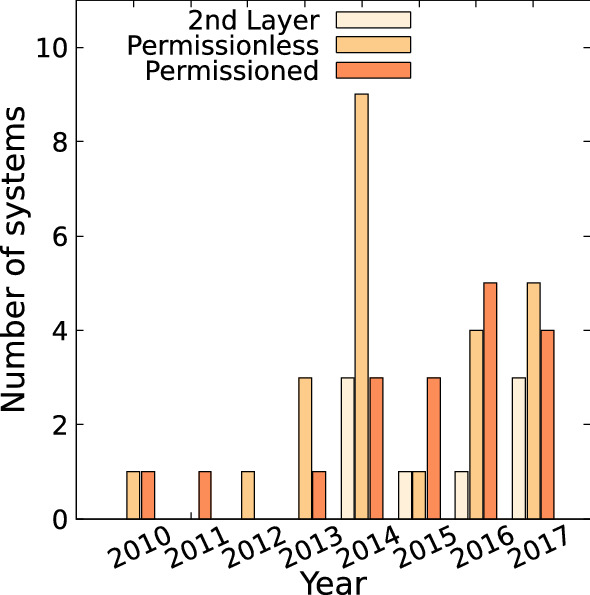
Number of Github repository creations of classified DLT systems for the clusters identified by k-means

Figure 11 depicts the number of new systems per year and cluster. The number of newly introduced systems peaked in 2014, when in total 15 of the 50 systems were introduced. This high number is mainly due to the introduction of permissionless systems. In recent years, the probability of introducing a permissioned or permissionless system is equal, while introducing a 2nd layer system has been lower.

The analysis concludes, that two key design choices in DLT systems are identified method-independently: *layering* and *participation level*. Moreover, *staking capability* and *cryptoeconomic complexity* are identified by MCA. The key design choices are not apparent in the taxonomy but are still captured by a combination of attribute values, which is an indication of the rich information the taxonomy can encode and explain. Hence, those findings support the explanatory capacity of the taxonomy as defined in earlier taxonomy theory [52]. Moreover, the combination of attribute values into key design choices identified by the analysis limits the system configuration options and as a result reduces modeling complexity of DLT systems at design phase.

### Summary of findings

The key findings of the performed experiments are summarized as follows:

- The proposed taxonomy (Fig. 2) is *useful*, as defined in earlier taxonomy literature [52]. In particular, the blockchain community validates the taxonomy as robust and comprehensive (on average $79\%$ expressiveness, Sect. 6.1). Moreover, the taxonomy is extensible (Sect. 6.1) and explanatory (Sect. 6.2), as found by analyzing the blockchain community feedback and applying machine learning methods on the classification. These findings also showcase the educational value of the proposed taxonomy.
- When compared with other viable and actively maintained DLT systems (Table 4 Supplementary Material), recent distributed computing contributions focus on a small subset of potential DLT system design configurations (e.g. Blockchain-based systems with no cryptoeconomic Token) (Sect. 4.5). The documentation of design choices in some of the contributions is found to be limited which hinders the understanding of the proposed systems functioning and which could result in a duplication of effort.
- The classification of 50 viable and actively maintained DLT systems is accepted by the blockchain community (on average $83.7\%$ acceptance over all components, Sect. 6.1.2).
- The quantitative analysis of the classification identifies four key design choices that structure the modeling complexity of DLT systems at design phase (Sect. 6.2). Each of these choices combines several attribute values and thus reduces the configuration complexity of DLT systems.

In a nutshell, the findings demonstrate that the contributions of this paper support system designers to systematically study and design DLT systems: The conceptual architecture and taxonomy map the space of possible system design configurations and thus assist researchers to position a system within the DLT landscape. For instance, the taxonomy can support the identification of blockchain parameters as required by the framework of Pavithran et al. [58]. Moreover, the classification reflects well the design configurations of existing DLT systems. Finally, the identified design choices provide new insights about influential and determining system elements and thus accelerate the design process. Hence, the contributions of this work can support the distributed computing community to (i) explore the design configuration space of DLT systems, (ii) to create novel applications and (iii) to document their contributions comprehensively.

## Design guideline for distributed ledgers

**Fig. 12:**
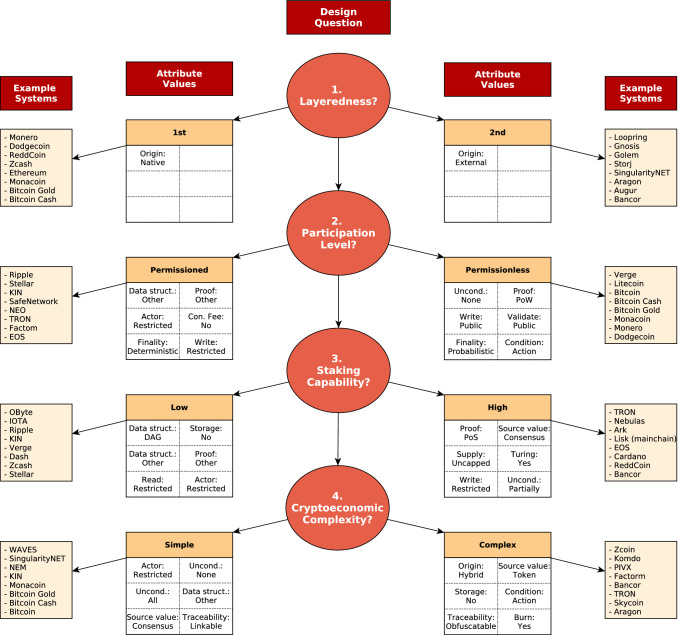
A design guideline of the key design choices in DLT systems, suggesting an order with which a designer may determine system configuration. The questions, attribute values, example systems, and order are a result of analysis conducted using real-world data and machine learning methods (Sect. 6.2). For each design decision identified via the MCA analysis, attribute values and the corresponding example systems that match best the respective design decision are illustrated

Based on the findings of the analysis (Sect. 6.3), a design guideline is derived (Fig. 12). The key design choices are determined quantitatively by applying machine learning algorithms on empirical data. The order is determined by the level of explained variance, as calculated by MCA.^17^ Each question corresponds to a binary design decision. For each decision, the six attribute values that contribute most to this design choice are illustrated. Moreover, for each choice, the systems that match best the attribute value configuration are depicted. Hence, this guideline structures the modeling complexity of DLT systems and can be used to accelerate the design phase by grouping together system design configurations. The ordering of design choices based on the explained variance in the observed DLT systems helps to differentiate existing DLT systems. As a result, the guideline can be used to provide new insights, support scientific novelty and business innovation. In particular, it can be used both for designing novel systems more effectively by providing default system configurations (Attribute values in Fig. 12) as well as to study DLT systems by illustrating similar systems (Example systems in Fig. 12).

The applicability of the guideline can be presented with the following: Designers determine the goals of the system and the constraints they have and then apply the guideline to identify a default system configuration. For instance, a designer develops a business model with a complex cryptoeconomic design (Decision 4 in Fig. 12). Due to time constraints, they go for building a second layer system (Decision 1 in Fig. 12). By looking at the suggested attribute values, the designers focus on attributes of the Action and Token component. In this case, amongst others, the designers are guided to consider token interactions (Source of value: Token) in their cryptoeconomic design. Moreover, by looking at the intersection of the systems falling under these design choices (Example Systems in Fig. 12), the Bancor and Aragon systems are identified as similar. Hence the guideline informs researchers about DLT systems sharing underlying commonalities.

In a nutshell, the derived guideline structures the modeling complexity of DLT systems at design phase and determines systems that share similar design choices. These design choices are systematically and rigorously determined using machine learning techniques and empirical data from existing viable and actively maintained DLT systems. Therefore such a guide can provide a more informed and tailored understanding of the DLT architectural elements, accelerate the design phase, prevent a duplication of effort and thus support the distributed computing and business community to innovate more efficiently.

## Conclusion and future work

This paper concludes that the evolving complexity of distributed ledgers can be better understood via a proposed taxonomy of DLT systems designed according to standards of state-of-the-art taxonomy theory [52]. To support such understanding, this paper contributes a systematic and rigorous classification of 50 viable and actively maintained DLT systems into the taxonomy using wisdom of the crowd and machine learning methods fed with real-world data. From that data a novel design guideline is derived that identifies key design choices that govern the complexity of distributed ledgers. The contributed guideline is a result of a novel data-driven methodology that structures the modeling complexity of DLT systems at design phase and thus can support the business and distributed computing community to innovate. Its value in education lies in better understanding and comparing the design of distributed ledgers.

Hence, the contributions of this paper can explain and provide new insights for researchers, practitioners and entrepreneurs about which choices in the DLT system design space have the highest impact, where there is room for innovation and which systems have competitive features or shared designs.

The results point to various avenues for future research. Firstly, the findings of this paper suggest that the taxonomy can be further extended with additional Action and Token attributes. Also, a component modeling the governance of the systems may become critical in deciding if a system has a decentralized organization (e.g. no trusted party). Secondly, although the taxonomy represents the current state of viable and actively maintained DLT systems, the proposed methods to evaluate its usefulness are general. Hence, future research can quantify with the introduced methodology the extent to which suggested extensions affect the usefulness of the proposed taxonomy. Thirdly, the initial cluster analysis demonstrates that key design choices can be derived quantitatively by analyzing empirical data of viable and actively maintained DLT systems. This suggests to extend the classification in future work (e.g. with Blockchain-as-a-Service systems such as Hyperledger Fabric [4]) and to apply different statistical methods to the data in order to validate and further identify key design choices. In particular, further design choices that illustrate token layouts such as the identified Staking Capability (Fig. 12) could facilitate the creation of novel incentives to address societal challenges [21, 41] (e.g, data and service management challenges in smart cities [3, 60]).

## Supplementary Information

Below is the link to the electronic supplementary material.

Supplementary material 1 (PDF 801 KB)

## Footnotes

1 Decentralized identities, owned and controlled by the individual represented through the identity.

2 Bitcoin uses a Nakamoto consensus, see Sect. 4.2.

3 In particular, paying a block reward (Sect. 4.4) and transaction fees (Sect. 4.2) to its consensus participants.

4 Faulty transactions of the same token to two different receivers.

5 Setup of fake identities to insert faulty information into the distributed ledger.

6 Usefulness is defined in Nickerson et al. [52] and formerly introduced in Sect. 5.2.2.

7 There are DLs that enable the execution of programmable scripts that are purposefully non-Turing-complete [87], e.g. in order to facilitate secure logic on financial transactions. These will currently be classified as “No”. Hence, in future work the Turing completeness attribute could be extended to the concept of Computational Capability which would take values in “None”, “Limited” and “Touring-complete”.

8 E.g. that malicious participants control less than one-third of the nodes of the consensus network [13].

9 These are systems not utilizing a native distributed ledger, as defined in Table 3 of the Supplementary Material.

10 A three-letter code identifying the token of a system.

11 Database of peer-reviewed literature, enabling full-text searches: https://www.elsevier.com/solutions/sciencedirect (last accessed: January 2021).

12 Searches over all article excluding references: https://dev.elsevier.com/tecdoc_sdsearch_migration.html (last accessed: May 2019).

13 Available at https://github.com (last accessed: January 2021).

14 In brackets are depicted the percentage for which this responds type accounts for the overall received comments. Please note, that the percentages do not add up to $100\%$ as a survey participant’s comments can account for more than one responds type.

15 Since the participant’s feedback the definition of the source of value has been revised. Please refer to Sect. 2 of the Supplementary Material

16 “Highly stable” clusters should yield values of 0.85 and above: https://rdrr.io/cran/fpc/man/clusterboot.html (last accessed: January 2021).

17 This proposed order is not strict and it represents which design choices have the larger impact on differentiating the existing DLT systems analyzed.

## References

[1] Abdi, H., Valentin, D.: Multiple correspondence analysis. In: Encyclopedia of Measurement and Statistics, vol. 2(4), pp. 651–657. SAGE, Thousand Oaks (2007)

[2] Aitzhan NZ, Svetinovic D (2016). Security and privacy in decentralized energy trading through multi-signatures, blockchain and anonymous messaging streams. IEEE Trans. Depend Secure Comput..

[3] Aloqaily M, Al Ridhawi I, Salameh HB, Jararweh Y (2019). Data and service management in densely crowded environments: challenges, opportunities, and recent developments. IEEE Commun. Mag..

[4] Androulaki, E., Barger, A., Bortnikov, V., Cachin, C., Christidis, K., De Caro, A., Enyeart, D., Ferris, C., Laventman, G., Manevich, Y., et al.: Hyperledger fabric: a distributed operating system for permissioned blockchains. In: Proceedings of the Thirteenth EuroSys Conference, pp. 1–15 (2018)

[5] Baars, D.: Towards self-sovereign identity using blockchain technology. Master’s thesis, University of Twente (2016)

[6] Babbitt, D., Dietz, J.: Crypto-economic design: a proposed agent-based modeling effort. Conference Talk. University of Notre Dame, Notre Dame, USA (2014)

[7] Bentov, I., Gabizon, A., Mizrahi, A.: Cryptocurrencies without proof of work. In: International Conference on Financial Cryptography and Data Security, pp. 142–157. Springer, Cham (2016)

[8] blog, A.: New horizons. http://blog.akasha.world/2017/11/14/new-horizons/. Accessed 21 Jan 2021

[9] Bonneau, J., Miller, A., Clark, J., Narayanan, A., Kroll, J.A., Felten, E.W.: Sok: Research perspectives and challenges for bitcoin and cryptocurrencies. In: 2015 IEEE Symposium on Security and Privacy (SP), pp. 104–121. IEEE (2015)

[10] Brown-Cohen, J., Narayanan, A., Psomas, A., Weinberg, S.M.: Formal barriers to longest-chain proof-of-stake protocols. In: Proceedings of the 2019 ACM Conference on Economics and Computation, pp. 459–473 (2019)

[11] Butean, A., Pournaras, E., Tara, A., Turesson, H., Ivkushkin, K.: Dynamic consensus: Increasing blockchain adaptability to enterprise applications. In: Computer Science On-line Conference, pp. 433–442. Springer, Cham (2020)

[12] Cachin, C., Vukolić, M.: Blockchain consensus protocols in the wild. arXiv preprint (2017). arXiv:1707.01873

[13] Castro, M., Liskov, B., et al.: Practical byzantine fault tolerance. In: OSDI, vol. 99, 173–186 (1999)

[14] Comuzzi, M., Unurjargal, E., Lim, C.: Towards a design space for blockchain-based system reengineering. In: International Conference on Advanced Information Systems Engineering, pp. 138–143. Springer, Cham (2018)

[15] Dash whitepaper: https://github.com/dashpay/dash/wiki/Whitepaper. Accessed 21 Jan 2021

[16] Davidson, S., De Filippi, P., Potts, J.: Economics of blockchain. SSRN Working Paper No 2744751. SSRN (2016). https://ssrn.com/abstract=2744751

[17] De Angelis, S., Aniello, L., Baldoni, R., Lombardi, F., Margheri, A., Sassone, V.: Pbft vs proof-of-authority: applying the cap theorem to permissioned blockchain. In: Italian Conference on Cybersecurity (2018)

[18] De Kruijff, J., Weigand, H.: Towards a blockchain ontology. Research report Tillburg University (2017)

[19] De La Rosa, J.L., Torres-Padrosa, V., El-Fakdi, A., Gibovic, D., Hornyák, O., Maicher, L., Miralles, F.: A survey of blockchain technologies for open innovation. In: Proceedings of the 4th Annual World Open Innovation Conference, pp. 14–15 (2017)

[20] Dhillon, V., Metcalf, D., Hooper, M.: Decentralized organizations. In: Blockchain Enabled Applications, pp. 47–66. Apress, Berkeley (2017). 10.1007/978-1-4842-3081-7_5

[21] Dierksmeier C, Seele P (2018). Cryptocurrencies and business ethics. J. Bus. Ethics.

[22] Dinh, T.T.A., Wang, J., Chen, G., Liu, R., Ooi, B.C., Tan, K.L.: Blockbench: A framework for analyzing private blockchains. In: Proceedings of the 2017 ACM International Conference on Management of Data, pp. 1085–1100 (2017)

[23] Dinh TTA, Liu R, Zhang M, Chen G, Ooi BC, Wang J (2018). Untangling blockchain: a data processing view of blockchain systems. IEEE Trans. Knowl Data Eng..

[24] Gonzlez JC, Garca-Daz V, Nez-Valdez ER, Gmez AG, Crespo RG (2020). Replacing email protocols with blockchain-based smart contracts. Clust. Comput.

[25] Greenacre MJ (1984). Correspondence Analysis.

[26] Greenacre, M., Pardo, R.: Multiple correspondence analysis of a subset of response categories. SSRN 847647 (2005)

[27] Greenacre M, Blasius J (2006). Multiple Correspondence Analysis and Related Methods.

[28] Hafid A, Hafid AS, Samih M (2020). Scaling blockchains: a comprehensive survey. IEEE Access.

[29] Hennig C (2007). Cluster-wise assessment of cluster stability. Comput. Stat. Data Anal..

[30] Iqbal R, Butt TA (2020). Safe farming as a service of blockchain-based supply chain management for improved transparency. Clust. Comput..

[31] Jain AK (2010). Data clustering: 50 years beyond k-means. Pattern Recognit. Lett..

[32] Jain AK, Maheswari S (2012). Survey of recent clustering techniques in data mining. Int. J. Comput. Sci. Manag. Res..

[33] Kakushadze Z, Russo RP (2018). Blockchain: data malls, coin economies, and keyless payments. J. Altern. Invest..

[34] Kalinin, K.P., Berloff, N.G.: Blockchain platform with proof-of-work based on analog Hamiltonian optimisers. arXiv preprint (2018). arXiv:1802.10091

[35] Kang J, Yu R, Huang X, Maharjan S, Zhang Y, Hossain E (2017). Enabling localized peer-to-peer electricity trading among plug-in hybrid electric vehicles using consortium blockchains. IEEE Trans. Ind. Inform..

[36] Kanwal T, Anjum A, Khan A (2020). Privacy preservation in e-health cloud: taxonomy, privacy requirements, feasibility analysis, and opportunities. Cluster Computing.

[37] Kappos, G., Yousaf, H., Maller, M., Meiklejohn, S.: An empirical analysis of anonymity in zcash. In: 27th $$\{$$USENIX$$\}$$ Security Symposium ($$\{$$USENIX$$\}$$ Security 18), pp. 463–477 (2018)

[38] Kewell B, Adams R, Parry G (2017). Blockchain for good?. Strateg. Change.

[39] Khalid U, Asim M, Baker T, Hung PC, Tariq MA, Rafferty L (2020). A decentralized lightweight blockchain-based authentication mechanism for IoT systems. Clust. Comput..

[40] Kiayias, A., Russell, A., David, B., Oliynykov, R.: Ouroboros: a provably secure proof-of-stake blockchain protocol. In: Annual International Cryptology Conference, pp. 357–388. Springer, Cham (2017)

[41] Kleineberg KK, Helbing D (2016). A “social bitcoin” could sustain a democratic digital world. Eur. Phys. J. Spec. Top..

[42] Korpela, K., Hallikas, J., Dahlberg, T.: Digital supply chain transformation toward blockchain integration. In: Proceedings Of The 50th Hawaii International Conference on System Sciences (2017)

[43] Kshetri, N., Voas, J.: Blockchain-enabled e-voting. pp. 95–99. IEEE Software (2018)

[44] Kshetri N (2018). Blockchain’s roles in meeting key supply chain management objectives. Int. J. Inf. Manag..

[45] Latif RMA, Farhan M, Rizwan O, Hussain M, Jabbar S, Khalid S (2020). Retail level blockchain transformation for product supply chain using truffle development platform. Clust. Comput..

[46] Lewenberg, Y., Sompolinsky, Y., Zohar, A.: Inclusive block chain protocols. In: International Conference on Financial Cryptography and Data Security, pp. 528–547. Springer, Cham (2015)

[47] Li H, Pei L, Liao D, Wang X, Xu D, Sun J (2020). BDDT: use blockchain to facilitate IoT data transactions. Clust. Comput..

[48] Manohar, A., Briggs, J.: Identity management in the age of blockchain 3.0. Association for Computing Machinery (2018)

[49] Mingxiao, D., Xiaofeng, M., Zhe, Z., Xiangwei, W., Qijun, C.: A review on consensus algorithm of blockchain. In: 2017 IEEE International Conference on Systems, Man, and Cybernetics (SMC), pp. 2567–2572. IEEE (2017)

[50] Moreno-Sanchez P, Ruffing T, Kate A (2017). Pathshuffle: credit mixing and anonymous payments for ripple. Proc. Privacy Enhancing Technol..

[51] Morisse, M.: Cryptocurrencies and bitcoin: charting the research landscape. In: Proceedings of Americas Conference on Information Systems (2015)

[52] Nickerson RC, Varshney U, Muntermann J (2013). A method for taxonomy development and its application in information systems. Eur. J. Inf. Syst..

[53] Norman G (2010). Likert scales, levels of measurement and the “laws” of statistics. Adv. Health Sci. Educ..

[54] Notheisen, B., Hawlitschek, F., Weinhardt, C.: Breaking down the blockchain hype–towards a blockchain market engineering approach. In: Proceedings of Americas Conference on Information Systems (2017)

[55] Notheisen, B., Willrich, S., Diez, M., Weinhardt, C.: Requirement-driven taxonomy development—a classification of blockchain technologies for securities post-trading. In: Proceedings of the 52nd Hawaii International Conference on System Sciences (2019)

[56] Okada, H., Yamasaki, S., Bracamonte, V.: Proposed classification of blockchains based on authority and incentive dimensions. In: 2017 19th International Conference on Advanced Communication Technology (ICACT), pp. 593–597. IEEE (2017)

[57] Pass, R., Shi, E.: Rethinking large-scale consensus. In: 2017 IEEE 30th Computer Security Foundations Symposium (CSF), pp. 115–129. IEEE (2017)

[58] Pavithran D, Shaalan K, Al-Karaki JN, Gawanmeh A (2020). Towards building a blockchain framework for IoT. Clust. Comput..

[59] Peterson, J., Krug, J., Zoltu, M., Williams, A.K., Alexander, S.: Augur: A decentralized oracle and prediction market platform Cryptoasset Report (2018)

[60] Pournaras E (2020). Proof of witness presence: blockchain consensus for augmented democracy in smart cities. J Parallel and Distributed Computing.

[61] Pouyanfar S, Tao Y, Tian H, Chen SC, Shyu ML (2019). Multimodal deep learning based on multiple correspondence analysis for disaster management. World Wide Web.

[62] Rauchs, M., Glidden, A., Gordon, B., Pieters, G.C., Recanatini, M., Rostand, F., Vagneur, K., Zhang, B.Z.: Distributed ledger technology systems: a conceptual framework. SSRN 3230013 (2018)

[63] Rendón E, Abundez I, Arizmendi A, Quiroz EM (2011). Internal versus external cluster validation indexes. International Journal of computers and communications.

[64] Ripple transacton cost. https://developers.ripple.com/transaction-cost.html. Accessed 21 Jan 2021

[65] Rosa, M., Barraca, J.P., Rocha, N.P.: Blockchain structures to guarantee logging integrity of a digital platform to support community-dwelling older adults. Clust. Comput. **23**, 1887–1898 (2020)

[66] Samuel, R.E.: A layered architectural approach to understanding distributed cryptographic ledgers. Issues Inf. Syst. **17**(IV), 222–226 (2016)

[67] Sankar, L.S., Sindhu, M., Sethumadhavan, M.: Survey of consensus protocols on blockchain applications. In: 2017 4th International Conference on Advanced Computing and Communication Systems (ICACCS), pp. 1–5. IEEE (2017)

[68] Sarfraz U, Alam M, Zeadally S, Khan A (2019). Privacy aware iota ledger: Decentralized mixing and unlinkable iota transactions. Computer Networks.

[69] Sarkintudu, S.M., Ibrahim, H.H., Abdwahab, A.B.: Taxonomy development of blockchain platforms: information systems perspectives. In: AIP Conference Proceedings, vol. 2016, p. 020130. AIP Publishing (2018)

[70] SiaHub: https://siahub.readme.io/reference. Accessed 21 Jan 2021

[71] Singh, N., Kumar, T., Vardhan, M.: Blockchain-based e-cheque clearing framework with trust based consensus mechanism. Clust. Comput. (2020). 10.1007/s10586-020-03163-6

[72] Steemit whitepaper: https://steem.io/steem-whitepaper.pdf. Accessed 21 Jan 2021

[73] Sukhwani, H., Martínez, J.M., Chang, X., Trivedi, K.S., Rindos, A.: Performance modeling of pbft consensus process for permissioned blockchain network (hyperledger fabric). In: 2017 IEEE 36th Symposium on Reliable Distributed Systems (SRDS), pp. 253–255. IEEE (2017)

[74] Sun, S.F., Au, M.H., Liu, J.K., Yuen, T.H.: Ringct 2.0: A compact accumulator-based (linkable ring signature) protocol for blockchain cryptocurrency monero. In: European Symposium on Research in Computer Security, pp. 456–474. Springer (2017)

[75] Tapscott, A., Tapscott, D.: How blockchain is changing finance. Harv. Bus. Rev.** 10**, 2–5 (2017)

[76] Tasca, P., Tessone, C.J.: A taxonomy of blockchain technologies: Principles of identification and classification. Ledger (2019). 10.5195/ledger.2019.140

[77] The Anatomy of a Transaction. https://domschiener.gitbooks.io/iota-guide/content/chapter1/transactions-and-bundles.html. (Accessed: 2021-01-21)

[78] Tian, H., Chen, S.C.: Mca-nn: Multiple correspondence analysis based neural network for disaster information detection. In: 2017 IEEE Third International Conference on Multimedia Big Data (BigMM), pp. 268–275. IEEE (2017)

[79] Tschorsch F, Scheuermann B (2016). Bitcoin and beyond: A technical survey on decentralized digital currencies. IEEE Communications Surveys & Tutorials.

[80] Tseng L, Wong L, Otoum S, Aloqaily M, Othman JB (2020). Blockchain for managing heterogeneous internet of things: A perspective architecture. IEEE Network.

[81] Vukolić, M.: Rethinking permissioned blockchains. In: Proceedings of the ACM Workshop on Blockchain, Cryptocurrencies and Contracts, pp. 3–7. ACM (2017)

[82] Wieninger, S., Schuh, G., Fischer, V.: Development of a blockchain taxonomy. In: 2019 IEEE International Conference on Engineering, Technology and Innovation (ICE/ITMC), pp. 1–9. IEEE (2019)

[83] Wu, L., Meng, K., Xu, S., Li, S., Ding, M., Suo, Y.: Democratic centralism: a hybrid blockchain architecture and its applications in energy internet. In: 2017 IEEE International Conference on Energy Internet (ICEI), pp. 176–181. IEEE (2017)

[84] Wüst, K., Gervais, A.: Do you need a blockchain? In: 2018 Crypto Valley Conference on Blockchain Technology (CVCBT), pp. 45–54. IEEE (2018)

[85] Xiong T, Wang S, Mayers A, Monga E (2012). Dhcc: Divisive hierarchical clustering of categorical data. Data Mining and Knowledge Discovery.

[86] Xu, X., Pautasso, C., Zhu, L., Gramoli, V., Ponomarev, A., Tran, A.B., Chen, S.: The blockchain as a software connector. In: Software Architecture (WICSA), 2016 13th Working IEEE/IFIP Conference on, pp. 182–191. IEEE (2016)

[87] Xu, X., Weber, I., Staples, M., Zhu, L., Bosch, J., Bass, L., Pautasso, C., Rimba, P.: A taxonomy of blockchain-based systems for architecture design. In: 2017 IEEE International Conference on Software Architecture (ICSA), pp. 243–252. IEEE (2017)

[88] Yeow K, Gani A, Ahmad RW, Rodrigues JJ, Ko K (2018). Decentralized consensus for edge-centric internet of things: A review, taxonomy, and research issues. IEEE Access.

[89] Zhang, K., Jacobsen, H.: Towards dependable, scalable, and pervasive distributed ledgers with blockchains. In: 2018 IEEE 38th International Conference on Distributed Computing Systems (ICDCS), pp. 1337–1346 (2018)

