# Supplementary material for: Decrypting distributed ledger design—taxonomy, classification and blockchain community evaluation
Source: Cluster Comput. 2021 Apr 24;25(3):1817–38. doi: 10.1007/s10586-021-03256-w (PMC9114082; doi:10.1007/s10586-021-03256-w)
Supplement: Supplementary file 1 — Supplementary material 1 (PDF 801 KB) [file 10586_2021_3256_MOESM1_ESM.pdf]

Noname manuscript No.
(will be inserted by the editor)

Supplemenatary Material to Decrypting Distributed Ledger Design

Taxonomy, Classification and Blockchain Community Evaluation

Mark C. Ballandies · Marcus M. Dapp · Evangelos Pournaras

the date of receipt and acceptance should be inserted later

Abstract Supplementary Material to Decrypting Distributed Ledger Design.

1 Survey Participants Invitation Email

Figure 1 depicts the email inviting participants to the survey.

2 Incorporating Blockchain Community Feedback

In the initial taxonomy and classification in the first phase of the survey, the underlying attribute was replaced with the on-chain underlying and off-chain underlying attributes to illustrate in finer detail the source of value of a token. In particular, in this version the un-

This work is supported by the Swiss National Science Foundation (grant no. 170226) for the European FLAG ERA project 'FuturICT 2.0 - Large scale experiments and simulations for the second generation of FuturICT' (https://futurict2.eu/) and the European Community's H2020 Program under the scheme 'ICT-10-2015 RIA', grant agreement #688364 'ASSET: Instant Gratification for Collective Awareness & Sustainable Consumerism' (http://www.asset-consumerism.eu).

Mark C. Ballandies
Computational Social Science
ETH Zurich
Tel.: +41 44 632 38 12
E-mail: mark.ballandies@ethz.ch

Marcus M. Dapp
Computational Social Science
ETH Zurich
Tel.: +41 44 632 03 46
E-mail: marcus.dapp@ethz.ch

Evangelos Pournaras
School of Computing
University of Leeds
E-mail: E.Pournaras@leeds.ac.uk

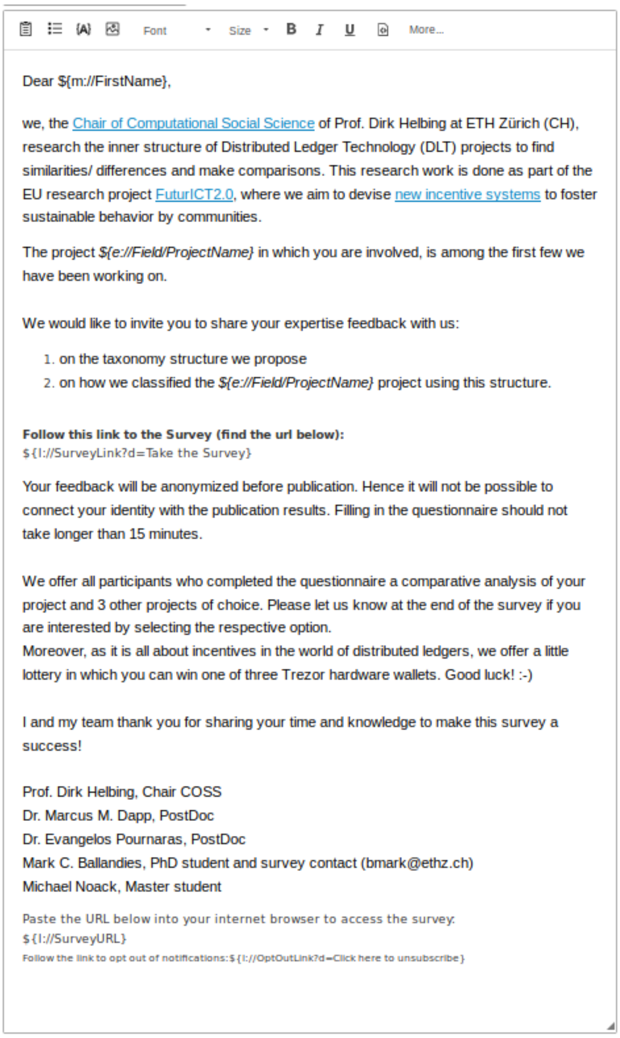
Font Size B I U More...

Dear ${m://FirstName},

we, the Chair of Computational Social Science of Prof. Dirk Helbing at ETH Zürich (CH), research the inner structure of Distributed Ledger Technology (DLT) projects to find similarities/ differences and make comparisons. This research work is done as part of the EU research project FuturICT2.0, where we aim to devise new incentive systems to foster sustainable behavior by communities.

The project *${e://Field/ProjectName}* in which you are involved, is among the first few we have been working on.

We would like to invite you to share your expertise feedback with us:

1. on the taxonomy structure we propose
2. on how we classified the *${e://Field/ProjectName}* project using this structure.

Follow this link to the Survey (find the url below):
${l://SurveyLink?d=Take the Survey}

Your feedback will be anonymized before publication. Hence it will not be possible to connect your identity with the publication results. Filling in the questionnaire should not take longer than 15 minutes.

We offer all participants who completed the questionnaire a comparative analysis of your project and 3 other projects of choice. Please let us know at the end of the survey if you are interested by selecting the respective option.
Moreover, as it is all about incentives in the world of distributed ledgers, we offer a little lottery in which you can win one of three Trezor hardware wallets. Good luck! :-)

I and my team thank you for sharing your time and knowledge to make this survey a success!

Prof. Dirk Helbing, Chair COSS
Dr. Marcus M. Dapp, PostDoc
Dr. Evangelos Pournaras, PostDoc
Mark C. Ballandies, PhD student and survey contact (bmark@ethz.ch)
Michael Noack, Master student

Paste the URL below into your internet browser to access the survey:
${l://SurveyURL}
Follow the link to opt out of notifications:${l://OptOutLink?d=Click here to unsubscribe}

Fig. 1 Survey participants invitation email.

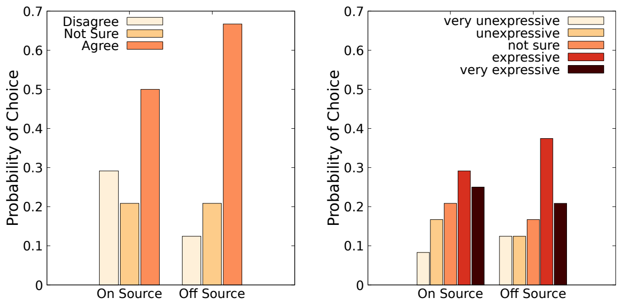

(a) Classification (N=24) (b) Expressiveness (N=24)

Fig. 2 Acceptance level of the classification and expressiveness of the On-chain and Off-chain underlying as perceived by survey participants.

derlying specifies the concrete object of value (e.g. storage or computation) and where it resides (on-chain or off-chain). Nevertheless, these attributes obtained the lowest acceptance level of all attributes (Figure 2). Detailed feedback regarding the attributes is summarized in the following and illustrated in the greater detail in Section 3. Namely, the main areas of disagreement or uncertainty are:

1. Not distinguishing between on-chain and off-chain underlying
2. Not distinguishing the underlying from services which can be assessed in the DLT system
3. Mixing up the underlying with the possibility to use a token as a currency
4. Considering future or past features of the DLT systems
5. Rejecting on-chain storage of hashes as storage

Based on this feedback, three changes have been made to the taxonomy.

First, in order to reduce the ambiguity regarding the difference between on-chain underlying, off-chain underlying and other services in the system (and hence addressing point one and two of the previous summary), the token underlying has been more clearly mapped to the components of the conceptual framework (Figure 1 in Paper). To achieve this, the off-chain and on-chain underlyings have been merged into one attribute and their values have been abstracted to include values from the framework where the underlying resides, as described in Section IV-D-6 of the paper. For instance, in the case of the Ethereum token, instead of expressing that the token grants access to the on-chain underlying *computation*, the token is now said to provide access to the *distributed ledger*, which in turn implies granting access to computation, because Ethereum has a Turing complete distributed ledger.

Second, to emphasize the option to store arbitrary data on distributed ledgers (e.g hashes), which, for instance, enables Bitcoin to function as the overarching infrastructure for other systems, the storage attribute has been added to the Distributed Ledger component. This addresses point number five.

Third, in order to address point number three, the transferability attribute has been added to the Token component, which emphasizes the possibility to use the token as a currency.

Finally, as the scope of the classification is to capture the current state of DLT systems and not future possible extensions or past configurations, point number four is not addressed.

The new transferability attribute is considered as the most expressive amongst all attributes by the community (Figure 6 in Paper). Moreover, the restructuring of the underlying attribute and the inclusion of the storage attribute improves the underlying assessment by the community significantly. These findings justify the modifications to the taxonomy. Also, the inclusion of new attributes into the taxonomy indicates that the taxonomy is extensible as defined in earlier taxonomy theory [2].

3 Detailed blockchain community feedback on previous underlying attributes

After the first two-month phase in which participants obtained the survey, the feedback has been analyzed which lead to changes in the taxonomy, as summarized in Section B. The detailed feedback is illustrated in the following.

When examining the comments provided by the participants who disagree with the classification of the on-chain underlying attribute[1], it is noteworthy that these respondents do not regard the on-chain storage of hashes as storage (22.2%). In some cases, respondents make contradictory comments about the on-chain value of their system token (11.1%). Some participants mix up the on-chain underlying of the token with the overall services that the DLT system provides (22.2%), which do not necessarily require to be accessed via the token. Some participants disagree with the classification because it does not consider plans to implement on-chain underlyings in the future (33.3%). Finally, some mix up the on-chain underlying with the off-chain underlying (11.1%).

[1] In brackets are depicted the percentage for which this responds type accounts for the overall disagreements. Please note, that the percentages do not add up to 100% as a survey participant could state several reasons for disagreement

Table 1 Attribute terminology improvements since the user study.

	Final term	Previous term	Reason for improvement
1	data structure	(DL) type	more precise
2	supply	supply property	more comprehensive
3	burn	burn property	more comprehensive
4	source of value	(on/ off chain) underlying	better mapping to conceptual architecture; more precise
5	finality	(consensus) type	more precise
6	creation condition	creation binding	more precise
7	unconditional creation	creation independence	more precise

Among respondents who express uncertainty, some do not distinguish between their current implementation of on-chain underlyings and possible or planned future implementations (50.0%), which are not in the scope of the classification. Other respondents mix up the possibility to use a token as a currency with its underlying (25.0%). Some state that the question is not formulated clearly enough (25.0%).

In the case of the off-chain underlying, a similar picture can be observed. In the following, the responses expressing disagreement and uncertainty are combined. Some respondents disagree with the classification because it does not consider past and future off-chain underlyings (30.0%). Some understood the off-chain underlying to be an exclusive right conferred by the token (10.0%). Moreover, as in the case of the on-chain underlying, some participants link the possibility to use the token as a currency to its underlying (20.0%). Some participants mix up on-chain and off-chain value (10.0%), others do not understand the question (10.0%) or do not respond (20.0%).

4 Changes in terminology

Table 1 depicts changes of attribute terminology that occurred since the user survey. The reasons for improvements are stated.

5 Machine Learning Analysis

Table 2 depicts the classified DLT systems in the new latent dimensions as identified by MCA (Section VI-B in Paper). The cluster associations are identified by k-means (Section VI-B in Paper).

Figure 3 illustrate the classified DLT systems in various combinations of the latent Dimensions, as identified by MCA (Section VI-B in Paper).

Figure 4 illustrates the Silhouette and Calinski-Harabasz scores when applying k-means for varying k on the classified systems (Section VI-B in Paper).

Table 2 DLT systems in the latent dimensions as identified by MCA. The clusters are identified by k-means.

id	DLT system	Cluster	Layer	Participation	Stakeability	Complexity
1	Aragon	1	0.06	0.81	0.71	0.64
2	Ark	0	0.97	0.69	0.96	0.31
3	Augur	1	0.08	0.84	0.68	0.49
4	Bancor	1	0.11	0.86	0.86	0.76
5	Bitcoin	2	0.97	0.96	0.48	0.22
6	Bitcoin Cash	2	0.97	0.96	0.48	0.22
7	Bitcoin Gold	2	0.97	0.96	0.48	0.22
8	BitShares	0	0.85	0.54	0.76	0.36
9	Byteball	0	0.93	0.52	0	0.6
10	Cardano	0	0.95	0.7	0.86	0.28
11	Dash	2	0.91	0.94	0.41	0.54
12	Decred	2	0.92	0.91	0.62	0.54
13	DigiByte	2	0.96	0.93	0.57	0.33
14	Dogecoin	2	0.99	0.95	0.58	0.24
15	EOS	0	0.97	0.43	0.89	0.5
16	Ethereum	2	0.97	0.87	0.76	0.35
17	Factom	0	0.88	0.43	0.73	0.89
18	Gnosis	1	0.01	0.74	0.63	0.57
19	Golem	1	0.03	0.76	0.55	0.24
20	IOTA	0	0.91	0.66	0.01	0.53
21	KIN	0	0.83	0.06	0.31	0.2
22	Komodo	2	0.92	0.95	0.54	0.95
23	Lisk-mainchain	0	0.94	0.65	0.96	0.36
24	Litecoin	2	0.97	0.96	0.48	0.22
25	Loopring	1	0	0.73	0.59	0.4
26	MOAC-MotherChain	2	0.96	0.93	0.57	0.33
27	Monacoin	2	0.97	0.96	0.48	0.22
28	Monero	2	1	0.96	0.42	0.47
29	Nebulas	0	0.96	0.56	1	0.34
30	NEM	0	0.93	0.63	0.76	0.16
31	NEO	0	0.84	0.35	0.71	0.46
32	Nexus	2	0.96	0.82	0.82	0.44
33	PIVX	2	0.88	0.71	0.67	0.91
34	Qtum	2	0.96	0.82	0.85	0.28
35	ReddCoin	2	0.97	0.86	0.86	0.31
36	Ripple	0	0.83	0	0.27	0.42
37	SafeNetwork	0	0.87	0.19	0.62	0.56
38	Siacoin	2	0.9	0.82	0.68	0.55
39	SingularityNET	1	0.04	0.7	0.48	0.01
40	Skycoin	2	0.86	0.45	0.42	0.72
41	Steem	0	0.93	0.56	0.81	0.6
42	Stellar	0	0.85	0.05	0.41	0.23
43	Storj	1	0.03	0.76	0.55	0.24
44	Stratis	2	0.95	0.86	0.76	0.45
45	Syscoin	2	0.89	0.9	0.49	0.64
46	TRON	0	0.89	0.39	1	0.76
47	Verge	2	0.97	1	0.39	0.37
48	Waves	0	0.91	0.47	0.77	0
49	Zcash	2	0.97	0.94	0.41	0.55
50	Zcoin	2	0.9	0.88	0.48	1

6 Cryptoeconomic Reasoning using Boolean Algebra

The taxonomy introduced in Section IV of the paper allows a number of widely used terms in the field of DLT systems to be more systematically defined by combining the values of specific attributes with operators from Boolean algebra. As demonstrated in Table 3, which features an illustrative subset of these terms, this method enables the delineation of terms such as permissioned/ permissionless blockchains, as well as asset/utility tokens. In particular, the latter pair has been identified by the Swiss Financial Market Supervisory Authority (FINMA) as important for determining whether a token should be classified as a security token. This is of interest to market participants because it has regulatory implications [1].

7 Classification

Tables 4-7 illustrate the classification of the 50 DLT systems in the four components.

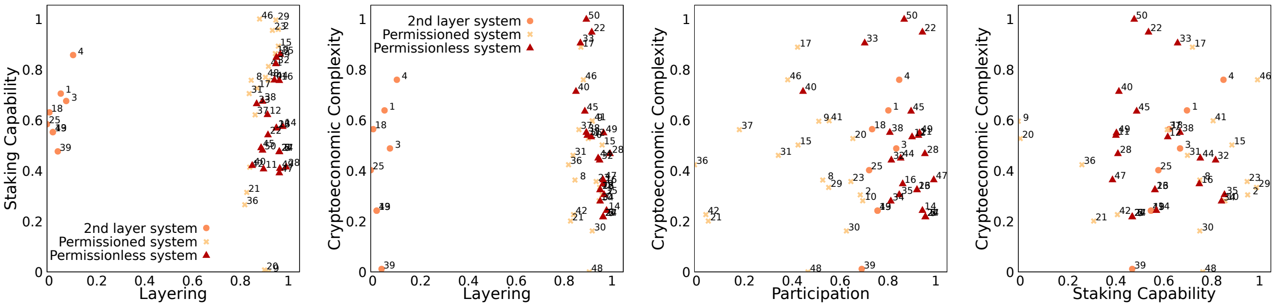

(a) Dimension 1 and 3 (b) Dimension 1 and 4 (c) Dimensions 2 and 4 (d) Dimension 3 and 4

Fig. 3 DLT systems in the latent dimensions, as identified by MCA. The labels are determined by the k-means clustering algorithm. The translation of the identifiers to DLT systems can be found in the Appendix of the paper.

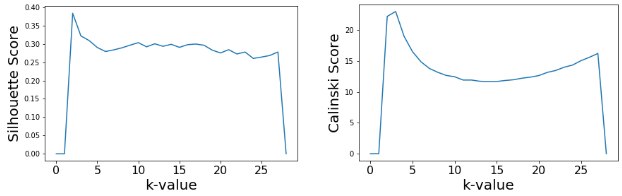

(a) Silhouette (b) Calinski-Harabasz

Fig. 4 Silhouette and Calinski-Harabasz score when applying kmeans for varying k on the original classification.

Table 3 Formal definitions of cryptoeconomic terms by reasoning using Boolean Algebra and the proposed taxonomy.

**System Term**	**Formal Definition**
Blockchain	*blockchain* type
1st layer	*native* ownership
2nd layer	*external* ownership
Permissioned	*restricted* write permission OR *restricted* validator permission
Permissionless	*public* write permission AND *public* validator permission
Public	⟨*permissionless DLT system*⟩ AND *public* actor permission
Private	⟨*permissioned DLT system*⟩ AND *restricted* actor permission
Privacy	*obfuscatable* traceability AND ⟨*public DLT System*⟩
Infrastructure	*yes* turing completeness OR *yes* storage
Blockchain-as-a-Service	$\nexists$ Action component attribute AND $\nexists$ Token component attribute
**Cryptoeconomic Term**	
Utility token	*distributed ledger* underlying OR *action* underlying
Asset token	*token* underlying OR *action* (physical good) underlying
Payment token	*yes* transferability

Table 4 Systems Classification according to the Distributed-Ledger component

ID	DLT system	Origin	Type	Address Traceability	Turing Completeness	Storage
1	Aragon	External (Ethereum)	-	-	-	-
2	Ark	Native	Blockchain	Linkable	No	Yes
3	Augur	External (Ethereum)	-	-	-	-
4	Bancor	External (Ethereum and EOS)	-	-	-	-
5	Bitcoin	Native	Blockchain	Linkable	No	Yes
6	Bitcoin Cash	Native	Blockchain	Linkable	No	Yes
7	Bitcoin Gold	Native	Blockchain	Linkable	No	Yes
8	BitShares	Native	Blockchain	Linkable	No	Yes
9	Byteball	Native	DAG	Obfuscatable	No	Yes
10	Cardano	Native	Blockchain	Linkable	No	Yes
11	Dash	Native	Blockchain	Obfuscatable	No	Yes
12	Decred	Native	Blockchain	Linkable	No	No
13	DigiByte	Native	Blockchain	Linkable	No	Yes
14	Dogecoin	Native	Blockchain	Linkable	No	Yes
15	EOS	Native	Blockchain	Linkable	Yes	Yes
16	Ethereum	Native	Blockchain	Linkable	Yes	Yes
17	Factom	Hybrid (Bitcoin)	Blockchain	Linkable	No	Yes
18	Gnosis	External (Ethereum)	-	-	-	-
19	Golem	External (Ethereum)	-	-	-	-
20	IOTA	Native	DAG	Linkable	No	No
21	KIN	Native	Other	Linkable	No	Yes
22	Komodo	Hybrid (Bitcoin)	Blockchain	Obfuscatable	No	Yes
23	Lisk (Mainchain)	Native	Blockchain	Linkable	No	Yes
24	Litecoin	Native	Blockchain	Linkable	No	Yes
25	Loopring	External (Ethereum, Qtum, NEO)	-	-	-	-
26	MOAC (MotherChain)	Native	Blockchain	Linkable	No	Yes
27	Monacoin	Native	Blockchain	Linkable	No	Yes
28	Monero	Native	Blockchain	Obfuscatable	No	Yes
29	Nebulas	Native	Blockchain	Linkable	Yes	Yes
30	NEM	Native	Blockchain	Linkable	No	Yes
31	NEO	Native	Blockchain	Linkable	Yes	Yes
32	Nexus	Native	Blockchain	Linkable	No	Yes
33	PIVX	Native	Blockchain	Obfuscatable	No	Yes
34	Qtum	Native	Blockchain	Linkable	Yes	Yes
35	ReddCoin	Native	Blockchain	Linkable	No	Yes
36	Ripple	Native	Other	Linkable	No	Yes
37	SafeNetwork	Native	Other	Linkable	Yes	Yes
38	Siacoin	Native	Blockchain	Linkable	No	Yes
39	SingularityNET	External (Ethereum)	-	-	-	-
40	Skycoin	Native	Blockchain	Obfuscatable	Yes	Yes
41	Steem	Native	Blockchain	Linkable	No	Yes
42	Stellar	Native	Other	Linkable	No	Yes
43	Storj	External (Ethereum)	-	-	-	-
44	Stratis	Native	Blockchain	Obfuscatable	No	Yes
45	Syscoin	Native	Blockchain	Obfuscatable	No	Yes
46	TRON	Native	Blockchain	Linkable	Yes	Yes
47	Verge	Native	Blockchain	Obfuscatable	No	Yes
48	Waves	Native	Blockchain	Linkable	Yes	Yes
49	Zcash	Native	Blockchain	Obfuscatable	No	Yes
50	Zcoin	Native	Blockchain	Obfuscatable	No	No

Table 5 Systems Classification according to the Consensus component

ID	DLT system	Finality	Proof	Write Permission	Validate Permission	Fee
1	Aragon	-	-	-	-	-
2	Ark	Probabilistic	PoS	Restricted	Restricted	Yes
3	Augur	-	-	-	-	-
4	Bancor	-	-	-	-	-
5	Bitcoin	Probabilistic	PoW	Public	Public	Yes
6	Bitcoin Cash	Probabilistic	PoW	Public	Public	Yes
7	Bitcoin Gold	Probabilistic	PoW	Public	Public	Yes
8	BitShares	Deterministic	PoS	Restricted	Public	Yes
9	Byteball	Deterministic	Other	Public	Restricted	Yes
10	Cardano	Probabilistic	PoS	Restricted	Restricted	Yes
11	Dash	Probabilistic	PoW	Public	Public	Yes
12	Decred	Probabilistic	Hybrid	Public	Public	Yes
13	DigiByte	Probabilistic	PoW	Public	Public	Yes
14	Dogecoin	Probabilistic	PoW	Public	Public	Yes
15	EOS	Deterministic	PoS	Restricted	Restricted	No
16	Ethereum	Probabilistic	PoW	Public	Public	Yes
17	Factom	Probabilistic	Other	Restricted	Restricted	No
18	Gnosis	-	-	-	-	-
19	Golem	-	-	-	-	-
20	IOTA	Probabilistic	PoW	Public	Restricted	No
21	KIN	Deterministic	Other	Restricted	Restricted	No
22	Komodo	Probabilistic	PoW	Public	Public	Yes
23	Lisk (Mainchain)	Probabilistic	PoS	Restricted	Restricted	Yes
24	Litecoin	Probabilistic	PoW	Public	Public	Yes
25	Loopring	-	-	-	-	-
26	MOAC (MotherChain)	Probabilistic	PoW	Public	Public	Yes
27	Monacoin	Probabilistic	PoW	Public	Public	Yes
28	Monero	Probabilistic	PoW	Public	Public	Yes
29	Nebulas	Deterministic	Pos	Restricted	Restricted	Yes
30	NEM	Probabilistic	PoS	Restricted	Restricted	Yes
31	NEO	Deterministic	Other	Restricted	Restricted	Yes
32	Nexus	Deterministic	Hybrid	Public	Public	Yes
33	PIVX	Deterministic	PoS	Public	Public	Yes
34	Qtum	Probabilistic	PoS	Public	Public	Yes
35	ReddCoin	Probabilistic	PoS	Public	Public	Yes
36	Ripple	Deterministic	Other	Restricted	Restricted	No
37	SafeNetwork	Deterministic	Other	Restricted	Restricted	No
38	Siacoin	Probabilistic	PoW	Public	Public	Yes
39	SingularityNET	-	-	-	-	-
40	Skycoin	Deterministic	Other	Public	Public	No
41	Steem	Deterministic	PoS	Restricted	Restricted	No
42	Stellar	Deterministic	Other	Restricted	Restricted	No
43	Storj	-	-	-	-	-
44	Stratis	Probabilistic	PoS	Public	Public	Yes
45	Syscoin	Probabilistic	PoW	Public	Public	Yes
46	TRON	Deterministic	PoS	Restricted	Restricted	No
47	Verge	Probabilistic	PoW	Public	Public	Yes
48	Waves	Deterministic	PoS	Restricted	Public	Yes
49	Zcash	Probabilistic	PoW	Public	Public	Yes
50	Zcoin	Probabilistic	PoW	Public	Restricted	Yes

Table 6 Systems Classification according to the Action component

ID	DLT system	Actor Permission	Read Permission	Fee
1	Aragon	Public	Public	Yes
2	Ark	Public	Public	No
3	Augur	Public	Public	Yes
4	Bancor	Public	Public	No
5	Bitcoin	Public	Public	No
6	Bitcoin Cash	Public	Public	No
7	Bitcoin Gold	Public	Public	No
8	BitShares	Public	Public	Yes
9	Byteball	Public	Restricted	No
10	Cardano	Public	Public	No
11	Dash	Public	Public	Yes
12	Decred	Public	Public	No
13	DigiByte	Public	Public	No
14	Dogecoin	Public	Public	No
15	EOS	Public	Restricted	No
16	Ethereum	Public	Public	No
17	Factom	Public	Public	Yes
18	Gnosis	Public	Public	Yes
19	Golem	Public	Public	Yes
20	IOTA	Public	Public	No
21	KIN	Restricted	Public	Yes
22	Komodo	Public	Public	No
23	Lisk (Mainchain)	Public	Public	Yes
24	Litecoin	Public	Public	No
25	Loopring	Public	Public	Yes
26	MOAC (MotherChain)	Public	Public	No
27	Monacoin	Public	Public	No
28	Monero	Public	Restricted	No
29	Nebulas	Public	Public	No
30	NEM	Public	Public	No
31	NEO	Public	Public	Yes
32	Nexus	Public	Public	No
33	PIVX	Public	Restricted	Yes
34	Qtum	Public	Public	No
35	ReddCoin	Public	Public	No
36	Ripple	Restricted	Restricted	Yes
37	SafeNetwork	Public	Public	No
38	Siacoin	Public	Public	Yes
39	SingularityNET	Restricted	Public	No
40	Skycoin	Public	Public	Yes
41	Steem	Public	Public	No
42	Stellar	Restricted	Public	Yes
43	Storj	Public	Public	Yes
44	Stratis	Public	Public	No
45	Syscoin	Public	Public	Yes
46	TRON	Public	Public	Yes
47	Verge	Public	Public	No
48	Waves	Restricted	Public	No
49	Zcash	Public	Restricted	No
50	Zcoin	Public	Public	No

Table 7 systems Classification according to the Token component

ID	DLT system	Supply Property	Burn Property	Transferability	Creation Condition	Unconditional Creation	Underlying
1	Aragon	Capped	Yes	transferable	Action	Partially	Action
2	Ark	Uncapped	No	transferable	Consensus	Partially	DL, Consensus
3	Augur	Capped	No	transferable	Action	Partially	Action
4	Bancor	Uncapped	Yes	transferable	Action	Partially	Token
5	Bitcoin	Capped	No	transferable	Consensus	None	DL
6	Bitcoin Cash	Capped	No	transferable	Consensus	None	DL
7	Bitcoin Gold	Capped	No	transferable	Consensus	None	DL
8	BitShares	Capped	Yes	transferable	None	All	DL, Consensus, Action
9	Byteball	Capped	No	transferable	None	All	DL
10	Cardano	Capped	No	transferable	Consensus	Partially	DL, Consensus
11	Dash	Capped	No	transferable	Both	None	DL, Action
12	Decred	Capped	No	transferable	Both	Partially	Consensus, Action
13	DigiByte	Capped	No	transferable	Consensus	Partially	DL
14	Dogecoin	Uncapped	No	transferable	Consensus	None	DL
15	EOS	Uncapped	No	transferable	Consensus	Partially	DL
16	Ethereum	Uncapped	No	transferable	Consensus	Partially	DL
17	Factom	Uncapped	Yes	transferable	Consensus	Partially	DL, Action
18	Gnosis	Capped	Yes	transferable	None	All	Token
19	Golem	Capped	No	transferable	None	All	Action
20	IOTA	Capped	No	transferable	None	All	None
21	KIN	Capped	No	transferable	None	All	DL, Action
22	Komodo	Capped	No	transferable	Both	Partially	DL, Token
23	Lisk (Mainchain)	Uncapped	No	transferable	Consensus	Partially	DL, Consensus
24	Litecoin	Capped	No	transferable	Consensus	None	DL
25	Loopring	Capped	Yes	transferable	None	All	Action
26	MOAC (MotherChain)	Capped	No	transferable	Consensus	Partially	DL
27	Monacoin	Capped	No	transferable	Consensus	None	DL
28	Monero	Uncapped	No	transferable	Consensus	None	DL
29	Nebulas	Uncapped	No	transferable	Consensus	Partially	DL, Consensus
30	NEM	Capped	No	transferable	None	All	DL, Consensus
31	NEO	Capped	No	transferable	None	All	Consensus, Action, Token
32	Nexus	Uncapped	No	transferable	Both	Partially	DL, Consensus
33	PIVX	Capped	Yes	transferable	Both	Partially	DL, Consensus, Action, Token
34	Qtum	Capped	No	transferable	Consensus	Partially	DL, Consensus
35	ReddCoin	Uncapped	No	transferable	Consensus	Partially	DL, Consensus
36	Ripple	Capped	Yes	transferable	None	All	DL, Action
37	SafeNetwork	Capped	Yes	transferable	Consensus	Partially	Action
38	Siacoin	Uncapped	Yes	transferable	Consensus	Partially	DL, Action
39	SingularityNET	Capped	No	transferable	None	All	Action
40	Skycoin	Capped	No	transferable	None	All	Token
41	Steem	Uncapped	No	transferable	Both	None	Token
42	Stellar	Uncapped	No	transferable	None	All	DL, Action
43	Storj	Capped	No	transferable	None	All	Action
44	Stratis	Uncapped	No	transferable	Consensus	Partially	DL, Consensus, A
45	Syscoin	Capped	No	transferable	Both	Partially	DL, Action
46	TRON	Uncapped	Yes	transferable	Consensus	Partially	DL, Token
47	Verge	Capped	No	transferable	Consensus	None	DL
48	Waves	Capped	No	transferable	None	All	DL, Consensus
49	Zcash	Capped	No	transferable	Consensus	Partially	DL
50	Zcoin	Capped	Yes	transferable	Both	Partially	DL, Token

8 Questionnaire

In the following the questionnaire as perceived by the survey participants of the second recruitment phase is displayed.

Intro

Dear ${e://Field/RecipientFirstName},

we, the Chair of Computational Social Science of Prof. Dirk Helbing at ETH Zürich (CH), research the inner structure of Distributed Ledger Technology (DLT) projects to find similarities/ differences and make comparisons. This research work is done as part of the EU research project FuturICT2.0, where we aim to devise new incentive systems to foster sustainable behavior by communities.

The project *${e://Field/ProjectName}* in which you are involved, is among the first few we have been working on.

We would like to invite you to share your expertise feedback with us:

1. on the taxonomy structure we propose
2. on how we classified the *${e://Field/ProjectName}* project using this structure.

Your feedback will be anonymized before publication. Hence it will not be possible to connect your identity with the publication results. Filling in the questionnaire should not take longer than 15 minutes.

We offer all participants who completed the questionnaire a comparative analysis of your project and 3 other projects of choice. Please let us know at the end of the survey if you are interested by selecting the respective option.
Moreover, as it is all about incentives in the world of distributed ledgers, we offer a little lottery in which you can win one of three Trezor hardware wallets. Good luck! :-)

I and my team thank you for sharing your time and knowledge to make this survey a success!

Prof. Dirk Helbing, Chair COSS
Dr. Marcus M. Dapp, PostDoc
Dr. Evangelos Pournaras, PostDoc
Mark C. Ballandies, PhD student and survey contact (bmark@ethz.ch)
Michael Noack, Master student

These page timer metrics will not be displayed to the recipient.

First Click: *0 seconds*

Last Click: *0 seconds*

Page Submit: *0 seconds*

Click Count: *0 clicks*

Demographics Block

Demographics

What is your role in the project?

- Project Lead
- Core/ Team Developer
- Team Member
- Community Developer
- Community Member
- Advisor
- other:

How many years did you work on DLT/ Blockchain projects?

- under one year
- one to three years
- more than three years

These page timer metrics will not be displayed to the recipient.
First Click: *0 seconds*
Last Click: *0 seconds*
Page Submit: *0 seconds*
Click Count: *0 clicks*

Introduction Block

Introduction

We describe DLT projects using four *components*: **Distributed Ledger**, **Consensus**, **Action** and **Token**.

Each component contains several *attributes. And each attribute can have* several *characteristics*.

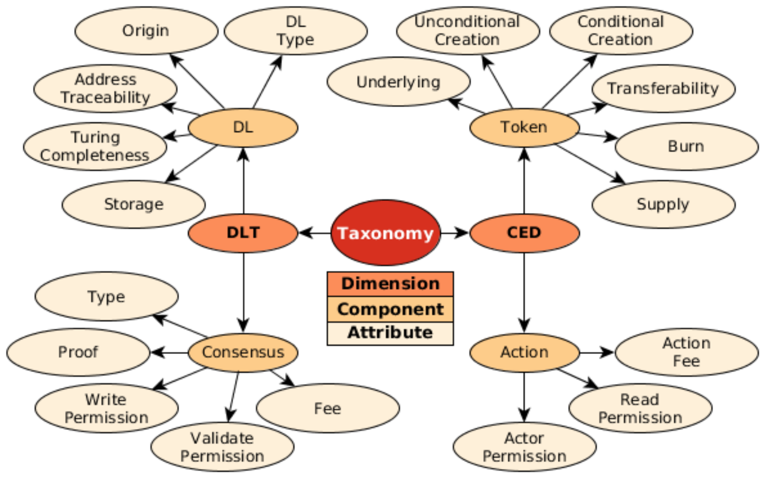

In the first part of the questionnaire we are interested in your opinion on the classification we performed for your project ${e://Field/ProjectName}.

These page timer metrics will not be displayed to the recipient.
First Click: *0 seconds*
Last Click: *0 seconds*
Page Submit: *0 seconds*
Click Count: *0 clicks*

Common DL Block

Part I
1. Distributed Ledger

The first *component* is the **Distributed Ledger**.

We define a distributed ledger as a distributed data structure, containing entries which serve as digital records of actions.

The five *attributes* are: **Origin**, **Type, Address Traceability, Turing Completeness** and **Storage**.

These page timer metrics will not be displayed to the recipient.
First Click: *0 seconds*
Last Click: *0 seconds*
Page Submit: *0 seconds*
Click Count: *0 clicks*

Part I
1. Distributed Ledger (1/5) - Origin

Origin describes who owns the distributed ledger.

The options are:
- **Native** if it is maintained by and for the project itself
- **External** if the project uses a distributed ledger from a different project
- **Hybrid** if the project uses both, a distributed ledger from a different project and natively developed distributed ledger

We have rated the **Origin** of the **Distributed Ledger** of **${e://Field/ProjectName}** as **${e://Field/DL_Origin}**. What is your opinion?

agree	disagree	not sure
○	○	○

If you disagree or are not sure please explain why:

These page timer metrics will not be displayed to the recipient.
First Click: *0 seconds*
Last Click: *0 seconds*
Page Submit: *0 seconds*
Click Count: *0 clicks*

Alternative DL Block

Part I
1. Distributed Ledger (2/4 - 5/5)

Because we rated the origin of your Distributed Ledger as External, the attributes **Type, Address**

Traceability, **Turing Completeness** and **Storage** were not classified by us for your project. For your information we explain them below.

Type describes the data structure of the distributed ledger.
The options are:
- **Blockchain**
- **Hashgraph**
- **Directed Acyclic Graph (DAG)**
- **Other**

Address Traceability describes the extent to which different transactions, which originate from or arrive at the same chain identity, can be linked together.
The options are:
- **Obfuscatable** if the distributed ledger has mechanisms in place to hide such links
- **Linkable** if links can be inferred with some computational effort.

Turing Completeness describes whether code can be executed on the distributed ledger.
The options are:
- **Yes** if it can
- **No** otherweise

Storage describes whether additonal data can be stored on the distributed ledger beyond the default transaction information.
The options are:
- **Yes** if additional data can be stored
- **No** otherweise

If you disagree or want to leave a comment please do:

These page timer metrics will not be displayed to the recipient.
First Click: *0 seconds*
Last Click: *0 seconds*
Page Submit: *0 seconds*
Click Count: *0 clicks*

Alternative C Block

Part I
2. Consensus

The second *component* is the **Consensus**.

We define Consensus as the mechanism of writing entries to the distributed ledger, adhering to a set of rules that all participants of the consensus will enforce when considering the validity of a entry and its containing actions.

The *five attributes* are: **Type**, **Proof**, **Write Permission**, **Validate Permission** and **Fee**.

These page timer metrics will not be displayed to the recipient.
First Click: *0 seconds*
Last Click: *0 seconds*
Page Submit: *0 seconds*
Click Count: *0 clicks*

Part I
2. Consensus (1/5 - 5/5)

The consensus is inherently linked to the utilized distributed ledger. Because your project does not utilize a native distributed ledger, the five attributes were not rated by us. For your information we explain them below.

Type describes whether the consensus is deterministic or probabilistic.
The options are:
- **Deterministic** if it is guaranteed that consensus can be reached in a finite period of time
- **Probabilistic** if consensus is found with some uncertainty

Proof describes the evidence which is used to achieve cosensus.
The options are
- **Proof-of-Work (PoW) if consensus is achieved usig processing power of computers**
- **Proof-of-Stake (PoS)**
- **Hybrid-PoW-PoS** is a combination of the previous two
- **Other** proving mechanisms

Write Permission describes who is allowed to write entries to the distributed ledger.
The options are:
- **Restricted** if participation is restricted
- **Public** otherwise

Validate Permission describes who is allowed to validate entries before they are written to the distributed ledger.

The options are:
- **Restricted** if participation is restricted
- **Public** otherwise

Fee describe whether participants in the consenus (i.e. writers, validators) are paid a fee for writing new entries to the dirtibuted ledger.
The options are:
- **Yes** if a fee is paid
- **No** otherwise

If you disagree or want to leave a comment please do:

These page timer metrics will not be displayed to the recipient.
First Click: *0 seconds*
Last Click: *0 seconds*
Page Submit: *0 seconds*
Click Count: *0 clicks*

Distributed Ledger Block

Part I
1. Distributed Ledger (2/5) - Type

Type describes the data structure of the distributed ledger.

The options are:
- **Blockchain**
- **Hashgraph**
- **Directed Acyclic Graph (DAG)**
- **Other**

We have rated the **Type** of the **Distributed Ledger** of **${e://Field/ProjectName}** as **${e://Field/DL_Type}**. What is your opinion?

agree	disagree	not sure
○	○	○

If you disagree or are not sure please explain why:

These page timer metrics will not be displayed to the recipient.
First Click: *0 seconds*
Last Click: *0 seconds*
Page Submit: *0 seconds*
Click Count: *0 clicks*

Part I
1. Distributed Ledger (3/5) - Address Traceability

Address Traceability describes the extent to which different transactions, which originate from or arrive at the same chain identity, can be linked together.

The options are:
- **Obfuscatable** if the distributed ledger has mechanisms in place to hide such links
- **Linkable** if links can be inferred with some computational effort

We rated the **Address Traceability** of the **Distributed Ledger** of **${e://Field/ProjectName}** as **${e://Field/DL_Traceability}**. ${e://Field/DL_Traceability} What is your opinion?

agree	disagree	not sure
○	○	○

If you disagree or are not sure please explain why:

These page timer metrics will not be displayed to the recipient.
First Click: *0 seconds*
Last Click: *0 seconds*
Page Submit: *0 seconds*
Click Count: *0 clicks*

Part I
1. Distributed Ledger (4/5) - Turing Completeness

Turing Completeness describes whether a Turing machine can be simulated by the distributed ledger.

The options are:
- **Yes** if it can
- **No** otherweise

We rated the **Turing Completenes** of the **Distributed Ledger** of **${e://Field/ProjectName}** as **${e://Field/DL_Turing}**. What is your opinion?

agree	disagree	not sure
○	○	○

If you disagree or are not sure please explain why:

These page timer metrics will not be displayed to the recipient.

First Click: *0 seconds*

Last Click: *0 seconds*

Page Submit: *0 seconds*

Click Count: *0 clicks*

Part I

1. Distributed Ledger (5/5) - Storage

Storage describes whether additional data can be stored on the distributed ledger beyond the default transaction information.

The options are:
- **Yes** if additional data can be stored
- **No**otherwise

We have rated the **Storage** of the **Distributed Ledger** of **${e://Field/ProjectName}** as **${e://Field/DL_Storage}**. What is your opinion?

agree	disagree	not sure
○	○	○

If you disagree or are not sure please explain why:

These page timer metrics will not be displayed to the recipient.
First Click: *0 seconds*
Last Click: *0 seconds*
Page Submit: *0 seconds*
Click Count: *0 clicks*

Consens Block

Part I
2. Consensus

The second *component* is the **Consensus**.

We define Consensus as the mechanism through which entries are written to the distributed ledger, while adhering to a set of rules that all participants enforce when an entry containing transactions is validated.

The five *attributes* are: **Type**, **Proof, Write Permission, Validate Permission** and **Fee**.

These page timer metrics will not be displayed to the recipient.
First Click: *0 seconds*
Last Click: *0 seconds*
Page Submit: *0 seconds*
Click Count: *0 clicks*

Part I
2. Consensus (1/5) - Type

Type describes whether the consensus is deterministic or probabilistic.

The options are:
- **Deterministic** if it is guaranteed that consensus can be reached in a finite period of time
- **Probabilistic** if consensus is found with some uncertainty

We have rated the **Type** of the **Consensus** of **${e://Field/ProjectName}** as **${e://Field/C_Type}**. What is your opinion?

agree | disagree | not sure

If you disagree or are not sure please explain why:

These page timer metrics will not be displayed to the recipient.
First Click: *0 seconds*
Last Click: *0 seconds*
Page Submit: *0 seconds*
Click Count: *0 clicks*

Part I
2. Consensus (2/5) - Proof

Proof describes the evidence used to achieve consensus.

The options are
- **Proof-of-Work (PoW)** if consensus is achieved using the processing power of computers
- **Proof-of-Stake (PoS) if** consesus is achieved through voting processes linked to (economic) power in the system
- **Hybrid-PoW-PoS** if is a combination of the previous two
- **Other** if another form of proof is required

We rated the **Proof** of the **Consensus** of **${e://Field/ProjectName}** as **${e://Field/C_Proof}**. What is your opinion?

agree | disagree | not sure

If you disagree or are not sure please explain why:

These page timer metrics will not be displayed to the recipient.
First Click: *0 seconds*
Last Click: *0 seconds*
Page Submit: *0 seconds*
Click Count: *0 clicks*

Part I
2. Consensus (3/5) - Write Permission

Write Permission describes who is allowed to write entries to the distributed ledger.

The options are:
- **Restricted** if participation is restricted
- **Public** otherwise

We have rated the **Write Permission** of the **Consensus** of **${e://Field/ProjectName}** as **${e://Field/C_WPermission}**. What is your opinion?

agree	disagree	not sure
○	○	○

If you disagree or are not sure please explain why:

These page timer metrics will not be displayed to the recipient.
First Click: *0 seconds*
Last Click: *0 seconds*
Page Submit: *0 seconds*
Click Count: *0 clicks*

Part I
2. Consensus (4/5) - Validate Permission

Validate Permission describes who is allowed to validate entries before they are written to the distributed ledger.

The options are:
- **Restricted** if participation is restricted
- **Public** otherwise

We have rated the **Validate Permission** of the **Consensus** of **${e://Field/ProjectName}** as **${e://Field/C_VPermission}**. What is your opinion?

agree	disagree	not sure
○	○	○

If you disagree or are not sure please explain why:

These page timer metrics will not be displayed to the recipient.
First Click: *0 seconds*
Last Click: *0 seconds*
Page Submit: *0 seconds*
Click Count: *0 clicks*

Part I
2. Consensus (5/5) - Fee

Fee describes whether participants of the consensus (i.e. writers, validators) are paid a fee for writing entries to the distributed ledger.

The options are:
- **Yes** if a fee is paid
- **No** otherwise

We have rated the **Fee** of the **Consensus** of **${e://Field/ProjectName}** as **${e://Field/C_Fees}**. What is your opinion?

agree	disagree	not sure
○	○	○

If you disagree or are not sure please explain why:

These page timer metrics will not be displayed to the recipient.
First Click: *0 seconds*
Last Click: *0 seconds*
Page Submit: *0 seconds*
Click Count: *0 clicks*

Action Block

Part I
3. Action

The third *component* is **Action**.

We define Action as one or more real-life activities, which can be digitally represented by the project as a transaction. One or more transactions form an entry on the Distributed Ledger.

The three *attributes* are: **Actor Permission, Read Permission** and **Fee**.

These page timer metrics will not be displayed to the recipient.
First Click: *0 seconds*
Last Click: *0 seconds*
Page Submit: *0 seconds*
Click Count: *0 clicks*

Part I
3. Action (1/3) - Actor Permission

Actor Permission describes who can perform an action.

The options are:
- **Restricted** if actors have to fulfill special requirements before performing actions (e.g. know-your-customer policies)
- **Public** if anyone can perform actions.

We have rated the **Actor Permission** of the **Action** of **${e://Field/ProjectName}** as **${e://Field/A_APermission}**. What is your opinion?

agree	disagree	not sure
○	○	○

If you disagree or are not sure please explain why:

These page timer metrics will not be displayed to the recipient.

First Click: *0 seconds*

Last Click: *0 seconds*

Page Submit: *0 seconds*

Click Count: *0 clicks*

Part I

3. Action (2/3) - Read Permission

Read Permission describes which actors can read the contents of transactions from the distributed ledger.

The options are:
- **Restricted** if permission is preconditioned (privacy coins restrict permission to the transaction participants)
- **Public** if permission is not restricted

We have rated the **Read Permission** of the **Action** of **${e://Field/ProjectName}** as **${e://Field/A_RPermission}**. What is your opinion?

agree	disagree	not sure
○	○	○

If you disagree or are not sure please explain why:

These page timer metrics will not be displayed to the recipient.
First Click: *0 seconds*
Last Click: *0 seconds*
Page Submit: *0 seconds*
Click Count: *0 clicks*

Part I
3. Action (3/3) - Fee

Fee describes whether an actor has to pay a fee for performing an action that is unrelated to the consensus.

The options are
- **Yes** if the actor has to pay
- **No** otherweise

We have rated the **Fee** of **Actions** of **${e://Field/ProjectName}** as **${e://Field/A_Fees}**. What is your opinion?

agree	disagree	not sure
○	○	○

If you disagree or are not sure please explain why:

These page timer metrics will not be displayed to the recipient.
First Click: *0 seconds*
Last Click: *0 seconds*
Page Submit: *0 seconds*
Click Count: *0 clicks*

Alternative T Block

Part I
4. Token

The last component of the taxonomy is the **Token**.

We define Token as a unit of value issued within a distributed ledger project and which can be used as a medium of exchange or unit of account.

The six attributes are:

- **Supply Property**
- **Burn Property**
- **Creation Binding**
- **Creation Independence**
- **On-chain source of value**
- **Off-chain source of value**

These page timer metrics will not be displayed to the recipient.
First Click: *0 seconds*
Last Click: *0 seconds*
Page Submit: *0 seconds*
Click Count: *0 clicks*

Part I

4. Token (1/6 - 6/6)

We found that your project does not use a Token, hence the six attributes are not specified. For your information we explain them below.

Supply Property describes the total quantity of tokens made available.
The options are:

- **Capped** if the total supply is bound by a finite number
- **Uncapped** otherwise

Burn Property describes whether token supply can be reduced by removing tokens..
The options are:

- **Yes** if removal is planned
- **No** otherwise

Creation Binding describes how the creation of **new** Tokens is bound to incentivize the Consensus and/or an Action.
The options are:

- **Consensus** if the creation is bound to the consenus
- **Action** if the creation is bound to an action
- **Both** if the creation is bound to the consensus and action
- **None** otherwise

Creation Independence describes the amount of **new** Tokens that are created **independent** of incentivizing the Consensus or Action.
The options are:

- **Partially** if some tokens are created independently

- **All** if all tokens are created independently (e.g. 100% pre-mined tokens)
- **None** otherwise

On-chain source of value describes to which extent the origin of value lies on the distributed ledger and out of what it constitutes.
The options are:
- **Governance** if the token gives access rights to governance mechanisms
- **Asset** if the token gives access to goods or commodities (i.e. to network fees paid in a different, project external token)
- **Consensus** if the token allows to participate in the Consensus of the project.
- **Identity** if the token gives access to an identity service (i.e. a domain name system)
- **Storage** if the token allows for storing data
- **Computation** if the token allows for the usage of computing power
- **Service** if the token gives access to another service than identity, storage or computation
- **None** if no on-chain source of value exists

Off-chain source of value describes to which extent the origin of value is independent of the distributed ledger and what it constitutes.
The options are:
- **Governance** if the token gives access rights to governance mechanisms
- **Asset** if the token gives access to a good or commodity
- **Storage** if the token allows for storing data
- **Computation** if the token allows for the usage of computing power
- **Service** if the token gives access to another service than storage or computation
- **None** if no off-chain source of value exists

If you disagree or want to leave a comment please do:

These page timer metrics will not be displayed to the recipient.
First Click: *0 seconds*
Last Click: *0 seconds*
Page Submit: *0 seconds*
Click Count: *0 clicks*

Token Block

Part I
4. Token

The last component of the taxonomy is the **Token**.

We define Token as a unit of value issued within a distributed ledger project and which can be used as a medium of exchange or unit of account.

The six attributes are: **Supply Property, Burn Property, Transferability, Conditional Creation, Unconditional Creation, Underlying**

We only focus on the **main Token** in case there are several in a project. In your case we classify the **${e://Field/TokenName}** token.

These page timer metrics will not be displayed to the recipient.
First Click: *0 seconds*
Last Click: *0 seconds*
Page Submit: *0 seconds*
Click Count: *0 clicks*

Part I
4. Token (1/6) - Supply Property

Supply Property describes the total quantity of tokens made available.

The options are:
- **Capped** if the total supply is bound by a finite number
- **Uncapped** otherwise

We have rated the **Supply Property** of the **Token** **${e://Field/TokenName}** as **${e://Field/T_Supply}**. What is your opinion?

agree ○ disagree ○ not sure ○

If you disagree or are not sure please explain why:

These page timer metrics will not be displayed to the recipient.
First Click: *0 seconds*
Last Click: *0 seconds*
Page Submit: *0 seconds*
Click Count: *0 clicks*

Part I
4. Token (2/6) - Burn Property

Burn Property describes whether the supply of token reduces when token units are removed.

The options are:
- **Yes** if removal is planned
- **No** otherwise

We have rated the **Burn Property** of the **Token** **${e://Field/TokenName}** as **${e://Field/T_Burned}**. What is your opinion?

agree ◯ disagree ◯ not sure ◯

If you disagree or are not sure please explain why:

These page timer metrics will not be displayed to the recipient.

First Click: *0 seconds*

Last Click: *0 seconds*

Page Submit: *0 seconds*

Click Count: *0 clicks*

Part I

4. Token (3/6) - Transferability

Off-chain source of value describes whether the ownership of a token unit can be changed.

The options are:
- **Transferable** if the token can be transfered
- **Non-transferable** otherwise

We have rated the Transferability of the **Token** **${e://Field/TokenName}** as **${e://Field/T_Transferability}**. What is your opinion?

agree ◯ disagree ◯ not sure ◯

If you disagree or are not sure please explain why:

Part I

4. Token (4/6) - Conditional Creation

Conditional Creation describes whether the creation of **new** token units is linked to the incentivization of the consensus mechanism and/ or an action.

The options are:

- **Consensus** if the creation is linked to the consensus mechanism
- **Action** if the creation is linked to an action
- **Both** if the creation is linked to the consensus mechanisms as well as an action
- **None** otherwise

We rated the **Conditional Creation** of the **Token** **${e://Field/TokenName}** as **${e://Field/T_CCreation}**. What is your opinion?

agree	disagree	not sure
○	○	○

If you disagree or are not sure please explain why:

These page timer metrics will not be displayed to the recipient.

First Click: *0 seconds*

Last Click: *0 seconds*

Page Submit: *0 seconds*

Click Count: *0 clicks*

Part I

4. Token (5/6) - Unconditional Creation

Unconditional Creation refers to the number of new token units that can be created which do not serve to incentivize the consensus mechanism or an action.

The options are:
- **Partial** if some tokens are created unconditionally
- **All** if all tokens are created unconditionally (e.g. 100% pre-mined tokens)
- **None** otherwise

We have rated the **Unconditional Creation** of the **Token** **${e://Field/TokenName}** as **${e://Field/T_UCreation}**. What is your opinion?

agree	disagree	not sure
○	○	○

If you disagree or are not sure please explain why:

These page timer metrics will not be displayed to the recipient.
First Click: *0 seconds*
Last Click: *0 seconds*
Page Submit: *0 seconds*
Click Count: *0 clicks*

Part I

4. Token (6/6) - Underlying

Underlying denotes the source of a token's value and what it consists of.

The options are:
- **Distributed Ledger** if the token grants access to the distributed ledger (e.g. if the token is required in order to use the storage or computing capacity of the distributed ledger)
- **Consensus** if the token grants access to the consesus mechanism
- **Action** if the token grants access to perform or receive actions or services
- **Token** if the token grants access to another token
- **None** if the token has no underlying

We have rated the **Underlying** of the **Token** **${e://Field/TokenName}** as **${e://Field/T_Underlying}**. What is your opinion?

agree	disagree	not sure
○	○	○

If you disagree or are not sure please explain why:

These page timer metrics will not be displayed to the recipient.
First Click: *0 seconds*
Last Click: *0 seconds*
Page Submit: *0 seconds*
Click Count: *0 clicks*

These page timer metrics will not be displayed to the recipient.
First Click: *0 seconds*
Last Click: *0 seconds*
Page Submit: *0 seconds*
Click Count: *0 clicks*

Taxonomy Block

Part II
1. Taxonomy

Now after you learned about the taxonomy on the concrete example of your project ${e://Field/ProjectName}, we would like you to give us some general feedback to the taxonomy.

These page timer metrics will not be displayed to the recipient.
First Click: *0 seconds*
Last Click: *0 seconds*
Page Submit: *0 seconds*
Click Count: *0 clicks*

Part II
2. Components

How expressive are the four *components* **Distributed Ledger, Consensus, Action and Token** to differentiate and classify DLT projects?

	very expressive	expressive	not sure	unexpressive	very unexpressive
Distributed Ledger	○	○	○	○	○
Consensus	○	○	○	○	○
Action	○	○	○	○	○
Token	○	○	○	○	○

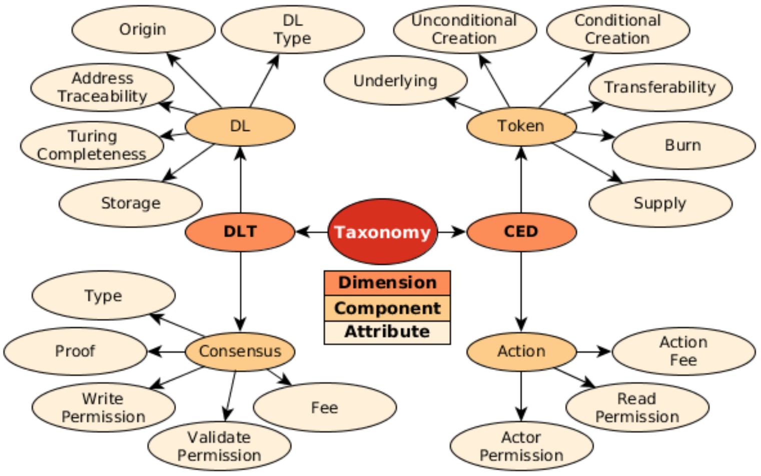

If you feel something is not covered or if you would like to suggest a different set of components please elaborate:

These page timer metrics will not be displayed to the recipient.

First Click: *0 seconds*

Last Click: *0 seconds*

Page Submit: *0 seconds*

Click Count: *0 clicks*

Part II

3. Attributes

You almost made it, this is the **last question!**

How expressive are the *attributes* to differentiate and classify DLT projects?

	very expressive	expressive	not sure	unexpressive	very unexpressive
**Distributed Ledger**					
Origin (in your case ${e://Field /DL_Origin})					
Type (in your case ${e://Field /DL_Type})					
Address Traceability (in your case ${e://Field /DL_Traceability})					
Turing Completeness (in your case ${e://Field /DL_Turing})					
Storage (in your case ${e://Field /DL_Storage})					
**Consensus**					
Type (in your case ${e://Field /C_Type})					
Proof (in your case ${e://Field /C_Proof})					
Write Permission (in your case ${e://Field /C_WPermission})					
Validate Permission (in your case ${e://Field /C_VPermission})					
Fee (in your case ${e://Field /C_Fees})					
**Action**					
Actor Permission (in your case ${e://Field /A_APermission})					
Read Permission (in your case ${e://Field /A_RPermission})					
Fee (in your case ${e://Field /A_Fees})					
**Token**					
Supply Property (in your case ${e://Field /T_Supply})					
Burn Property (in your case ${e://Field /T_Burned})					
Transferability (in your case ${e://Field /T_Transferability})					
Conditional Creation (in your case ${e://Field /T_CCreation})					
Unconditional Creation (in your case ${e://Field /T_UCreation})					
Underlying (in your case ${e://Field					

If you feel something is not covered or would like to suggest a different set of attributes please elaborate:

These page timer metrics will not be displayed to the recipient.
First Click: *0 seconds*
Last Click: *0 seconds*
Page Submit: *0 seconds*
Click Count: *0 clicks*

Block 6

Thank you very much! This is the end of the questionnaire. Feel free to leave some extra comments:

Are you interested in the comparative analysis of your project and 3 other projects of your choice?

Yes	No
○	○

Please state the three projects (if a project has not been analyzed, a different project will be given to you).

These page timer metrics will not be displayed to the recipient.
First Click: *0 seconds*
Last Click: *0 seconds*
Page Submit: *0 seconds*
Click Count: *0 clicks*

References

1. FINMA, E.F.: FINMA publishes ICO guidelines. https://www.finma.ch/en/news/2018/02/20180216-mm-ico-wegleitung/. (Accessed: 2018-07-27)
2. Nickerson, R.C., Varshney, U., Muntermann, J.: A method for taxonomy development and its application in information systems. European Journal of Information Systems **22**(3), 336–359 (2013)
